# Integrating polyphenols and exercise in cancer prevention, treatment, and rehabilitation: cellular and molecular mechanisms linking the injury–recovery–musculoskeletal resilience axis

**DOI:** 10.3389/fonc.2026.1801640

**Published:** 2026-07-10

**Authors:** Sen Lin, Mengrui Bi

**Affiliations:** 1 The Department of Police Command and Tactical, Zhejiang Police College, Zhejiang, Hangzhou, China; 2 School of Sports and Arts, Tangshan Maritime Institute, Tangshan, China

**Keywords:** cancer, collagen peptides, muscle regeneration, polyphenols, sports injury prevention

## Abstract

Cancer progression, therapeutic interventions, and rehabilitation profoundly affect musculoskeletal integrity, impair tissue resilience, and compromise recovery, leading to functional decline and reduced quality of life. Increasing evidence suggests that combining exercise with polyphenol supplementation exerts synergistic effects in preventing tissue injury, mitigating treatment-related damage, and promoting functional recovery. This review integrates current findings on the cellular and molecular mechanisms by which polyphenols such as curcumin, resveratrol, quercetin, and green tea catechins, modulate oxidative stress, inflammation, apoptosis, autophagy, and anabolic signaling in muscle, bone, and cardiac tissues. Exercise induces mechanical loading and metabolic adaptations that activate PI3K/Akt/mTOR, MAPK/ERK, AMPK, and Nrf2 pathways, enhancing protein synthesis, mitochondrial biogenesis, and redox-adaptive capacity. Polyphenols further reinforce these effects by suppressing NF-κB and pro-inflammatory cytokines, modulating BDNF/CREB in the nervous system, and attenuating chemotherapy- or radiotherapy-induced tissue damage. Together, these interventions support musculoskeletal resilience, accelerate recovery, and optimize rey -30habilitation outcomes. By elucidating these mechanistic pathways, this review provides a framework for evidence-based integrative strategies to enhance functional preservation and repair in cancer prevention, treatment, and rehabilitation.

## 1 Introduction

Sports participation, whether at recreational, competitive, or elite levels, offers substantial benefits for physical conditioning, psychological well-being, and social engagement. Nevertheless, athletic performance is often threatened by injuries that compromise tissue integrity, delay training cycles, and reduce overall career longevity (1). Epidemiological data indicate that a large proportion of athletes experience at least one injury during their active years, and in some sports, the rate can reach more than 81 injuries per 1000 competitors at international events (2). Musculoskeletal damage, particularly to muscles, ligaments, tendons, and bones, accounts for the majority of these cases and frequently results in prolonged absences from training and competition (3). In elite athletes, such time loss is not only a medical concern but also carries psychological and economic implications for the individual and the sporting organization. Despite advances in biomechanics, protective equipment, and conditioning programs, the global incidence of sports-related injuries has not markedly declined (4). Recovery and rehabilitation remain complex processes involving both physiological and behavioral components. Traditional approaches emphasize physiotherapy, load management, and pharmacological interventions, yet growing evidence highlights nutrition as a critical determinant of tissue resilience, inflammatory balance, and recovery efficiency (5). Adequate nutritional intake influences every stage of healing, from the inflammatory and repair phases to the remodeling of musculoskeletal structures, through coordinated regulation of energy availability, protein synthesis, immune function, and redox balance (6).

Recent systematic reviews have underscored that nutritional support should be individualized according to injury type, severity, and metabolic demands during rehabilitation (7). When energy intake is insufficient, the catabolic response intensifies, leading to delayed collagen synthesis, increased inflammatory signaling, and muscle atrophy. Conversely, balanced energy provision with sufficient macronutrients accelerates tissue regeneration and mitigates secondary complications such as sarcopenia or fat accumulation during immobilization (8). Protein and amino acid intake, particularly leucine-rich or collagen-derived peptides, play a central role in maintaining muscle mass and activating anabolic pathways in injured tissues (9, 10). Evidence also supports the complementary role of omega-3 polyunsaturated fatty acids in reducing pro-inflammatory eicosanoid production, thereby promoting a microenvironment conducive to healing (11). In addition to macronutrients, micronutrients and bioactive compounds have attracted increasing attention for their contribution to musculoskeletal integrity. Vitamins C and E act as antioxidants that regulate oxidative stress during the inflammatory phase, while vitamin D and calcium are essential for bone mineralization and the prevention of stress fractures (12–14). Trace minerals such as zinc, magnesium, and iron further support enzymatic processes required for collagen cross-linking and oxygen transport (15). Polyphenols, especially flavonoids, are reported to enhance vascular perfusion and attenuate oxidative damage in recovering tissues (16). Moreover, probiotics may influence immune-muscle communication through gut–muscle axis mechanisms, improving protein absorption and modulating inflammatory responses (17). Beyond the musculoskeletal system, nutritional factors also appear to affect neurological recovery following sport-related concussions. Mild traumatic brain injury initiates a cascade of metabolic disturbances characterized by mitochondrial dysfunction, oxidative stress, and impaired glucose utilization (18). Specific nutrients, such as omega-3 fatty acids, sulfur-containing amino acids, antioxidants, and certain minerals, may mitigate these disturbances by restoring membrane fluidity and supporting antioxidant defenses (19). These observations collectively indicate that nutritional interventions exert systemic effects extending beyond localized tissue repair. The significance of nutrition extends across all ages and disciplines, from youth to professional athletes. In younger populations, the combination of rapid growth, high training volumes, and inadequate dietary intake increases vulnerability to both acute and overuse injuries (20, 21). Deficiencies in energy, protein, calcium, vitamin D, and iron can impair bone mineralization and muscle adaptation, thereby heightening the risk of stress fractures, ligamentous injuries, and delayed recovery. Accordingly, early nutritional education and food literacy represent essential preventive strategies for adolescents engaged in high-intensity sports (22). The dynamic interplay between exercise-induced stress, inflammation, and nutrition forms the foundation of modern sports traumatology and functional nutrition. Current frameworks emphasize a multidisciplinary approach integrating nutritional science with biomechanics, physiotherapy, and psychology (23). This integration is particularly vital as the metabolic cost of tissue repair demands coordinated regulation of energy balance, nutrient timing, and micronutrient availability (24). For example, the controlled combination of carbohydrates and proteins in post-exercise recovery has been shown to optimize glycogen resynthesis and stimulate muscle protein synthesis, supporting the athlete’s return to training readiness (25).

In parallel, cancer represents a major global health burden characterized by dysregulated proliferation, chronic inflammation, and disruption of cellular signaling networks. In this context, physical exercise and dietary interventions, particularly those rich in polyphenols, have emerged as promising non-pharmacological strategies for cancer prevention, treatment support, and rehabilitation. Regular physical activity reduces the incidence and progression of cancers such as breast, colorectal, and prostate through mechanisms including immune modulation, reduced systemic inflammation, improved metabolic regulation, and suppression of oncogenic signaling pathways (26).

Polyphenols, biologically active constituents prevalent in phytogenic edibles such as Camellia sinensis (green tea), various berries, Curcuma longa (turmeric), and Vitis vinifera (grapes), demonstrate redox-modulating, anti-inflammatory, and epigenetic regulatory properties that directly influence key processes such as oxidative stress, apoptosis, autophagy, and immune regulation (27). Rather than acting through isolated pathways, polyphenols and exercise converge on shared molecular targets, including NF-κB (nuclear factor kappa-light-chain-enhancer of activated B cells), Nrf2 (nuclear factor erythroid 2-related factor 2), AMPK (AMP-activated protein kinase), and SIRT1 (sirtuin 1), which are central to mitochondrial function, inflammation control, and cellular adaptation (28). This convergence provides a mechanistic basis for their synergistic effects on tissue repair, metabolic regulation, and musculoskeletal resilience.

The objective of this review is to furnish a thorough synthesis of contemporary evidence regarding the cellular and molecular mechanisms through which polyphenols and physical exercise interact to impact cancer prevention, treatment, and rehabilitation, with a specific emphasis on the injury–recovery–musculoskeletal resilience continuum. Specifically, this review focuses on how these interventions regulate oxidative stress, inflammation, apoptosis, and tissue remodeling within an integrated mechanistic framework. It investigates the synergistic effects of physical exercise and polyphenol consumption on tumor advancement, skeletal muscle preservation, bone health, cardiac safeguarding, and neural integrity within the framework of cancer and its associated therapies. By amalgamating molecular insights with translational and clinical perspectives, this review aspires to inform evidence-based methodologies for enhancing musculoskeletal resilience, expediting recovery, and ameliorating functional outcomes. Additionally, it delineates deficiencies in mechanistic, clinical, and nutritional research, thereby offering a strategic framework for future inquiries aimed at optimizing integrated lifestyle interventions within oncology. Importantly, the present review does not treat sports injury and cancer rehabilitation as separate biological contexts. Instead, exercise-related tissue repair is used as a mechanistic reference model to interpret cancer-associated tissue vulnerability. In oncology, musculoskeletal dysfunction is rarely caused by mechanical injury alone. It develops through the convergence of tumor-driven systemic inflammation, treatment-induced mitochondrial injury, endocrine disruption, impaired anabolic signaling, cachexia, sarcopenia, and reduced regenerative capacity (29–32). Therefore, the injury–recovery–musculoskeletal resilience axis proposed here is intended to describe a broader biological continuum in which cancer progression and anticancer therapies induce a chronic injury-like state, while exercise and polyphenol-rich nutritional interventions may support recovery by modulating immune-metabolic balance, mitochondrial homeostasis, redox signaling, and anabolic responsiveness (30, 33, 34). This cancer-centered interpretation is particularly relevant for patients exposed to chemotherapy, radiotherapy, hormonal therapy, or androgen deprivation therapy, in whom skeletal muscle loss, fatigue, frailty, and impaired rehabilitation capacity are major determinants of clinical outcome.

## 2 Conceptual framework: the injury–recovery–musculoskeletal resilience axis

**Figure 1:**
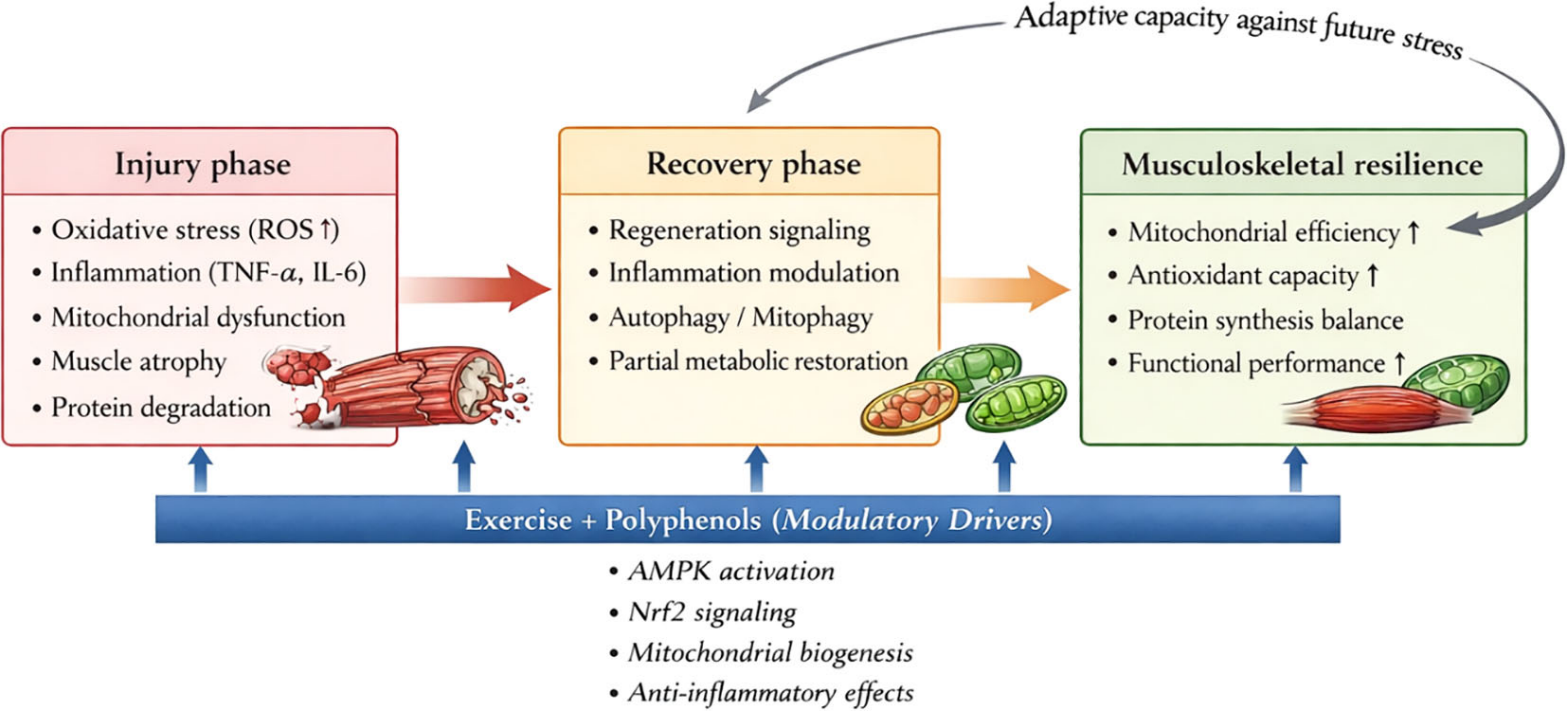
Conceptual illustration of the injury–recovery–musculoskeletal resilience continuum and the modulatory role of exercise and polyphenols.

To provide a coherent conceptual foundation for the present review, the “injury–recovery–musculoskeletal resilience axis” is introduced as an integrative framework describing the dynamic relationship between tissue damage, repair processes, and long-term functional adaptation in cancer-related conditions. Rather than being viewed as a linear sequence, this axis is better understood as a continuous and interdependent process through which biological systems respond to internal and external stressors, including tumor progression, therapeutic interventions, metabolic disruption, and systemic inflammation (**Figure 1**). Within this framework, injury is defined as the initial structural and functional disturbance induced by cancer and its treatment. This stage is characterized by alterations such as oxidative stress, mitochondrial dysfunction, inflammation, impaired protein homeostasis, and muscle atrophy, which have been widely reported in cancer-associated tissue dysfunction (32, 35). These processes collectively compromise cellular integrity and reduce the capacity of musculoskeletal tissues to maintain normal physiological function. The recovery phase encompasses the coordinated biological responses that aim to restore homeostasis following injury. This includes activation of regenerative signaling pathways, modulation of inflammatory responses, repair of damaged cellular components, and partial restoration of metabolic balance. However, in cancer settings, recovery processes are often incomplete or dysregulated due to persistent systemic stress, treatment-related toxicity, and impaired anabolic signaling.

Within this framework, cancer-associated musculoskeletal injury should be understood as a systemic and persistent biological condition rather than a localized mechanical lesion. Tumor progression and anticancer therapies create a chronic stress environment characterized by elevated inflammatory mediators, mitochondrial dysfunction, oxidative stress, endocrine alterations, impaired protein turnover, and reduced regenerative capacity (30–32, 36). These processes reduce the ability of skeletal muscle to respond to anabolic stimuli, a phenomenon commonly described as anabolic resistance (31, 37). Under physiological conditions, mechanical loading, amino acid availability, insulin/IGF-1 signaling, and androgen-dependent pathways converge on PI3K/Akt/mTOR signaling to promote protein synthesis, satellite-cell activity, mitochondrial and structural remodeling. In aging and cancer, however, this coordinated response becomes progressively blunted. Normal regenerative signals such as IGF-1/Akt/mTOR activation are attenuated, while catabolic pathways driven by chronic NF-κB activation, cytokine signaling, ubiquitin–proteasome activity, autophagy imbalance, mitochondrial stress, and ROS accumulation become dominant (30–32, 37). Consequently, the transition from injury to recovery is delayed, and the capacity to establish long-term musculoskeletal resilience is compromised.

A further layer of regulation involves endocrine and androgen-dependent signaling. Androgens contribute to skeletal muscle trophism through androgen receptor-mediated genomic and non-genomic mechanisms, including interactions with IGF-1 signaling, MAPK/ERK, Akt, Rac1, focal adhesion kinase, and cytoskeletal remodeling pathways (38, 39). Recent evidence indicates that androgen receptor expression declines in aged skeletal muscle and that androgen receptor cooperation with filamin A may protect skeletal muscle cells from oxidative stress-induced senescence (39). This is highly relevant to oncology because androgen decline, endocrine therapy, androgen deprivation therapy, chronic inflammation, and cachexia can converge to weaken anabolic signaling and accelerate sarcopenic remodeling (30, 38, 39). In parallel, aging, chronic inflammation, and cancer-related metabolic stress may impair satellite-cell function, reducing the ability of muscle to regenerate effectively after injury, inactivity, or treatment-induced damage (40). Thus, musculoskeletal resilience in cancer should be viewed as the integrated outcome of immune-inflammatory control, mitochondrial homeostasis, endocrine/anabolic competence, redox homeodynamics, and regenerative cell function. Importantly, ROS should not be interpreted only as damaging by-products of mitochondrial dysfunction. Although sustained ROS accumulation contributes to oxidative injury, proteolysis, inflammation, apoptosis, and impaired muscle regeneration, transient and compartmentalized ROS signals are also involved in transcriptional adaptation, mitochondrial biogenesis, antioxidant defense, and cellular remodeling (41, 42). This distinction is important for the present framework because exercise-, inflammation-, hormone-, and nutrient-dependent signals may influence recovery not only through antioxidant defense but also through redox-sensitive transcriptional and epigenetic mechanisms. For example, Perillo et al. showed that hormonally regulated ROS oscillations participate in estrogen-induced transcriptional cycling, while IKKα-dependent phosphorylation of histone H3 at serine 10 helps coordinate chromatin regulation and preserve DNA integrity (42). Although this evidence derives from estrogen receptor-positive breast cancer cells, it provides a useful conceptual bridge for oncology rehabilitation by showing that redox signaling can contribute to adaptive transcriptional regulation when properly coordinated. Therefore, the therapeutic goal should not be complete ROS suppression, but restoration of redox homeodynamics, in which chronic oxidative stress is limited while adaptive redox signaling is preserved.

The concept of musculoskeletal resilience extends beyond short-term recovery and refers to the ability of tissues to adapt, withstand, and function effectively under repeated or chronic stress conditions. This adaptive state is associated with improvements in mitochondrial efficiency, enhanced antioxidant defense systems, optimized protein turnover, and functional reinforcement of muscle and connective tissues (43, 44). Resilience therefore represents a higher-order integration of structural stability and functional capacity over time. Importantly, exercise and polyphenol-rich nutritional strategies can be positioned within this axis as modulatory factors that influence each stage of the process. Exercise is primarily associated with the activation of pathways related to energy metabolism, mitochondrial biogenesis, and neuromuscular adaptation, whereas polyphenols contribute to the regulation of oxidative stress, inflammatory signaling, and cellular protection mechanisms (45, 46). Their combined effects are therefore hypothesized to facilitate the transition from injury toward more efficient recovery and, ultimately, enhanced resilience. This framework is intended to serve as a unifying perspective through which the mechanistic and clinical evidence discussed in the following sections can be interpreted, enabling a more structured understanding of how biological processes and interventions interact across cancer prevention, treatment, and rehabilitation contexts. Within this conceptual framework, the evidence can be organized into five interconnected mechanistic modules: inflammatory control, redox homeodynamics, mitochondrial homeostasis, anabolic–catabolic balance, and receptor-mediated tissue remodeling. These modules are not independent. NF-κB-driven inflammation can amplify mitochondrial ROS production and proteolysis; mitochondrial dysfunction can reinforce inflammatory signaling and reduce exercise tolerance; impaired IGF-1/Akt/mTOR signaling can limit protein synthesis despite adequate nutrient availability; and receptor-mediated pathways such as EGFR, MAPK/ERK, and PI3K/Akt can connect growth factor signaling to proliferation, inflammation, migration, and tissue repair. This network-based interpretation allows exercise and polyphenol-rich nutrition to be understood as context-dependent regulators of biological system behavior rather than as isolated interventions acting on single pathways (30, 47, 48).

## 3 Methodological framework of the review

This article was developed as a structured narrative review aimed at integrating mechanistic, preclinical, and clinical evidence on the combined roles of polyphenols and exercise in cancer prevention, treatment, and rehabilitation. Particular emphasis was placed on the injury, recovery, and musculoskeletal resilience axis, which serves as a unifying framework across the different sections of this review. To ensure a coherent and comprehensive coverage of the topic, the literature was organized into key thematic domains, including the pathophysiological basis of tissue injury, the role of polyphenol-rich functional foods, the effects of exercise across different cancer-related conditions, underlying molecular signaling pathways, and translational considerations for clinical practice. This structure allowed for the integration of findings across different biological scales, from cellular mechanisms to functional outcomes. A structured literature search was conducted using major scientific databases, including PubMed, Web of Science, Scopus, and Google Scholar. Search terms were selected based on the scope of the review and included combinations of keywords such as “polyphenols,” “exercise,” “cancer,” “rehabilitation,” “muscle injury,” “oxidative stress,” “inflammation,” “mitochondrial dysfunction,” “fatigue,” “cachexia,” and “musculoskeletal resilience,” along with specific compounds such as “curcumin,” “resveratrol,” “quercetin,” and “catechins.” Additional relevant studies were identified through manual screening of reference lists in key articles.

Studies were selected based on their relevance to the interaction between polyphenol-related signaling, exercise-induced adaptations, and cancer- or treatment-associated tissue dysfunction. Experimental studies, animal models, clinical trials, and observational studies were considered when they provided mechanistic insight or translational relevance. Studies that were not directly related to oncology, did not address exercise or polyphenol mechanisms, or lacked relevance to musculoskeletal or functional outcomes were not included in the synthesis. Given the heterogeneity of the available literature in terms of study design, intervention protocols, and outcome measures, a formal systematic analysis was not performed. Instead, the evidence was synthesized using a narrative approach, with particular attention to recurring biological pathways and converging mechanisms. These included oxidative stress, inflammation, mitochondrial regulation, apoptosis, autophagy, and anabolic signaling, which consistently emerged as central mediators of tissue injury and recovery. This methodological approach was adopted to balance mechanistic depth with clinical relevance, while allowing the integration of diverse forms of evidence across prevention, treatment, and rehabilitation contexts in cancer.

## 4 Pathophysiological basis of sports injuries

Sports injuries are biological disruptions caused by acute trauma or repetitive mechanical stress that exceed the structural or functional tolerance of musculoskeletal tissues (49). They encompass a wide spectrum, from minor microtears to severe ruptures or fractures, and are classically categorized as acute (single high-load incident) or chronic/overuse (repeated sub-thresholdstress). Both types initiate overlapping cascades of tissue damage, inflammation, and repair (50). Epidemiological data consistently indicate that skeletal muscle is the tissue most frequently affected in sport, followed by tendons, ligaments, cartilage, and bone. Muscle injuries alone account for approximately 30% of all athletic injuries, especially in high-speed or contact disciplines (51). This vulnerability arises from the interplay of mechanical loading, metabolic stress, and neuromuscular control, particularly during eccentric contractions or fatigue. Overuse injuries develop when cumulative microdamage exceeds the tissue’s regenerative capacity, leading to maladaptive remodeling and degeneration (52, 53). Importantly, sports injuries extend beyond local structural disruption and involve coordinated neuroendocrine, immune, and metabolic responses (54). These integrated responses provide the biological basis for targeted nutritional and therapeutic interventions.

### 4.1 Mechanical and structural basis of tissue damage

The initial phase of any sports injury involves mechanical disruption of tissue integrity. During high-intensity activity, the applied mechanical stress, stretching, compression, or shear, can exceed the tensile or viscoelastic limits of the involved structures (55). In skeletal muscle, excessive stretch during an active contraction leads to sarcomere inhomogeneity, where weaker sarcomeres are overstretched and disrupted, producing local contracture knots and membrane rupture (56). Loss of sarcolemmal integrity permits calcium influx, activating proteases such as calpains and initiating proteolytic and necrotic pathways (57, 58). Similarly, tendons and ligaments experience micro-failures of collagen fibrils under repetitive cyclic loading. Loss of sarcolemmal integrity permits calcium influx, activating proteases such as calpains and initiating proteolytic and necrotic pathways (59, 60). Bone, though more rigid, undergoes repetitive stress microfractures when osteoclastic resorption temporarily surpasses osteoblastic formation (61). Mechanotransduction pathways, including integrin signaling and focal adhesion kinase (FAK), regulate whether mechanical stimuli promote adaptation or injury (62). Thus, sports injuries can be viewed as a disruption of load–repair balance, where mechanical demand exceeds the tissue’s capacity for maintenance and regeneration (63). When loading exceeds repair capacity, adaptive remodeling is replaced by pathological degeneration, resulting in chronic conditions such as tendinopathy and stress fractures (64).

### 4.2 Cellular responses to mechanical stress

Mechanical strain translates into biochemical signaling within affected cells, initiating cascades that regulate inflammation, metabolism, and regeneration. Following sarcolemmal rupture, calcium influx triggers activation of calpains and phospholipases, leading to cytoskeletal degradation and mitochondrial dysfunction (65). Damaged muscle fibers release damage-associated molecular patterns (DAMPs) such as HMGB1 and ATP, which act as alarmins to recruit immune cells and activate toll-like receptor (TLR) pathways (66, 67). In the extracellular matrix (ECM), mechanical distortion stimulates fibroblasts and tenocytes through mechanosensitive ion channels and integrins, activating downstream pathways such as MAPK, NF-κB, and PI3K/Akt (68). These pathways coordinate both catabolic and anabolic processes, promoting matrix metalloproteinase (MMP)-mediated ECM degradation and, subsequently, collagen synthesis through transforming growth factor-β (TGF-β) signaling (69). These responses are temporally regulated. Within hours of injury, muscle fibers undergo necrosis or apoptosis, while neutrophils and macrophages infiltrate the damaged tissue to remove debris (70). The balance between pro- and anti-inflammatory cytokines (e.g., TNF-α, IL-6, IL-10) determines progression toward resolution or persistent inflammation (71, 72). Mechanical stress is also coupled to metabolic signaling. Mitochondrial dysfunction, ATP depletion, and reactive oxygen species (ROS) accumulation amplify injury signaling, linking structural damage to metabolic imbalance (73).

### 4.3 Inflammatory cascade and immune modulation

Inflammation is a central and tightly regulated component of sports injury pathology. Following tissue disruption, increased vascular permeability facilitates the infiltration of plasma proteins and immune cells into the damaged site. Neutrophils are among the earliest responders, releasing ROS and proteolytic enzymes to clear necrotic tissue. While essential, excessive neutrophil activity can exacerbate secondary damage through bystander effects (74–76). Subsequently, macrophages dominate the lesion milieu and exhibit dynamic phenotypic changes. The early M1 phenotype produces pro-inflammatory cytokines such as IL-1β and TNF-α, while the later M2 phenotype promotes resolution via IL-10, TGF-β, and growth factors that stimulate angiogenesis and myogenesis. The transition from M1 to M2 macrophages is essential for effective healing, and its disruption may result in fibrosis or chronic inflammation (77, 78).

Recent studies highlight the involvement of innate lymphoid cells, T cells, and mast cells in regulating repair. For example, T regulatory cells (Tregs) modulate macrophage polarization and promote satellite-cell differentiation (79). Additionally, cytokine networks integrate with endocrine and metabolic axes, linking immune activation to systemic stress responses mediated by cortisol and catecholamines (80). Inflammation also mediates cross-talk among multiple tissues. Adipokines (e.g., leptin, adiponectin) influence cytokine production, whereas myokines such as IL-6 and irisin exert systemic metabolic effects, integrating local injury with whole-body adaptation (81, 82). When unresolved, inflammation becomes detrimental. Persistent cytokine elevation sustains fibroblast activation and ECM deposition, resulting in fibrotic scar formation that compromises elasticity and force transmission (83). In the context of cancer, the inflammatory response extends beyond local tissue injury and becomes a systemic driver of musculoskeletal dysfunction. Tumor progression and anticancer therapies are often associated with sustained elevation of pro-inflammatory cytokines such as TNF-α and IL-6, which can directly interfere with muscle protein homeostasis and cellular signaling. These cytokines have been shown to activate catabolic pathways, impair mitochondrial function, and disrupt muscle regeneration processes, thereby contributing to muscle weakness and functional decline. Moreover, the tumor-associated inflammatory microenvironment can exacerbate mitochondrial stress and promote maladaptive processes such as excessive mitophagy, further compromising muscle integrity (84). Therefore, therapeutic strategies aim to modulate rather than suppress inflammation, preserving its regenerative role while preventing chronic pathological activation.

### 4.4 Oxidative stress and mitochondrial dysregulation

Exercise- and injury-related oxidative stress represents another key component of the pathophysiological response. While transient increases in reactive oxygen and nitrogen species (ROS/RNS) are part of normal signaling, excessive accumulation following injury leads to oxidative damage of lipids, proteins, and DNA (43). Mitochondria act as both a major source and target of ROS. Calcium overload and impaired electron transport chain (ETC) function enhance superoxide generation, which subsequently induces mitochondrial permeability transition and activation of cell-death pathways (85, 86). Oxidative stress also interacts with inflammatory signaling, as elevated ROS levels amplify NF-κB activation and sustain cytokine production (87). Conversely, mild oxidative signals can activate adaptive responses via Nrf2-dependent antioxidant gene expression, underscoring a biphasic role of ROS, harmful at high levels but beneficial in moderation (88). Energy metabolism is profoundly altered during this period. ATP depletion limits ion pump activity, aggravating calcium dysregulation and cytoskeletal collapse (45). Simultaneously, local hypoxia stabilizes HIF-1α, promoting angiogenesis and metabolic reprogramming toward glycolysis, processes required for the transition from inflammation to tissue repair (89, 90). Antioxidant systems, including superoxide dismutase, catalase, glutathione peroxidase, and non-enzymatic molecules like vitamins C and E, play protective roles, but their effectiveness depends on adequate nutritional support and timing. When oxidative stress persists, it contributes to delayed healing, mitochondrial fragmentation, and impaired contractile recovery (91, 92).

In cancer-related conditions, oxidative stress represents a critical downstream consequence of both tumor metabolism and treatment-induced cellular damage, with direct implications for skeletal muscle integrity. Excessive accumulation of ROS disrupts mitochondrial function by impairing electron transport chain activity, reducing ATP production, and increasing membrane permeability. This mitochondrial dysfunction not only compromises energy supply required for muscle contraction and repair, but also promotes the activation of cell death pathways, including apoptosis and ferroptosis (93). Importantly, when mitochondrial damage exceeds the capacity of quality control mechanisms, such as mitophagy, dysfunctional organelles accumulate and further amplify oxidative stress. This leads to a progressive decline in muscle cell viability and contributes to structural deterioration and loss of contractile function. These processes are particularly relevant in cancer patients, where persistent oxidative stress can accelerate muscle wasting, reduce functional capacity, and impair recovery following treatment (94). Therefore, regulating oxidative stress through both endogenous defense mechanisms and nutritional modulation is critical to maintain mitochondrial function and accelerate recovery.

It is important to emphasize that ROS have a dual biological role in this framework. In cancer-associated cachexia, therapy-induced toxicity, and chronic inflammation, sustained ROS accumulation can damage lipids, proteins, mitochondrial DNA, and nuclear DNA, thereby promoting mitochondrial dysfunction, proteolysis, apoptosis, ferroptosis, and impaired muscle regeneration (32, 36, 95). However, ROS also function as tightly regulated signaling mediators. Transient ROS generation during exercise contributes to mitochondrial biogenesis, antioxidant enzyme induction, insulin sensitivity, and skeletal-muscle adaptation, whereas excessive ROS suppression, particularly through high-dose antioxidant supplementation, may attenuate some ROS-dependent exercise adaptations (41, 48, 96). Thus, the therapeutic goal is not complete elimination of ROS, but restoration of redox homeodynamics, in which damaging oxidative stress is limited while adaptive redox signaling is preserved. This broader interpretation is further supported by evidence showing that hormonally regulated ROS oscillations can participate in transcriptional cycling and chromatin regulation. In estrogen receptor-positive MCF-7 cells, IKKα-dependent phosphorylation of histone H3 at serine 10 contributed to cyclical ROS production during estrogen-induced transcription and helped preserve DNA integrity. When IKKα activity was inhibited, oxidized guanine accumulation and apoptotic signaling increased, indicating that ROS-linked transcriptional cycling and epigenetic control may be protective when properly coordinated (42). Although this evidence derives from estrogen-responsive breast cancer cells rather than skeletal muscle, it provides an important conceptual bridge for the present review: exercise-, inflammation-, hormone-, and nutrient-dependent redox signals may influence cellular adaptation not only through antioxidant defense pathways but also through chromatin remodeling, transcriptional regulation, and genome-maintenance mechanisms.

This redox-sensitive framework is particularly relevant when interpreting polyphenol-based interventions during exercise and cancer rehabilitation. Sustained or excessive ROS accumulation contributes to lipid peroxidation, protein oxidation, mitochondrial DNA injury, inflammatory amplification, apoptosis, ferroptosis, and impaired muscle regeneration. However, transient and compartmentalized ROS generation also supports adaptive signaling during exercise and tissue remodeling through pathways involved in mitochondrial biogenesis, antioxidant enzyme induction, insulin sensitivity, and skeletal-muscle adaptation. These responses involve redox-sensitive signaling networks such as AMPK, PGC-1α, MAPK, and Nrf2-related pathways (97, 98). Therefore, the therapeutic objective should not be complete ROS elimination, but restoration of redox homeodynamics, in which chronic oxidative injury is limited while adaptive redox signaling is preserved.

This distinction is essential when interpreting polyphenol use during exercise-based rehabilitation. Nrf2 activation, for example, should be considered an adaptive cytoprotective response rather than simply an antioxidant endpoint. Acute exercise-induced ROS can activate Nrf2-dependent transcriptional programs that increase endogenous antioxidant capacity and cellular stress tolerance (96). Conversely, excessive ROS suppression by high-dose or poorly timed antioxidant supplementation may reduce the redox signals required for physiological training adaptation. Human and mechanistic studies indicate that high-dose antioxidant supplementation can attenuate some exercise-induced improvements in insulin sensitivity, endogenous antioxidant defense, mitochondrial biogenesis, and skeletal-muscle remodeling (48, 99). Accordingly, polyphenol-rich nutrition should be framed as a strategy for redox modulation rather than indiscriminate ROS scavenging.

### 4.5 Muscle regeneration and satellite-cell dynamics

Following the initial inflammatory phase, skeletal-muscle recovery depends on the activation and coordination of satellite cells, the resident myogenic stem cells located beneath the basal lamina (100). Once activated by cytokines, growth factors, and mechanical cues, these quiescent cells enter the cell cycle, proliferate as myoblasts, and fuse to form new myofibers or repair existing ones. This process is modulated by transcription factors such as Pax7, MyoD, and Myf5, which orchestrate lineage specification and fusion (100, 101). Effective regeneration requires a balance between myogenesis and fibrosis. Excessive inflammation or mechanical instability favors fibroblast proliferation and ECM deposition, which may limit fiber regeneration and elasticity (102). The transition toward repair is mediated by growth factors such as insulin-like growth factor-1 (IGF-1) and hepatocyte growth factor (HGF), which enhance satellite-cell activation and differentiation (103, 104). Cross-talk between immune cells and satellite cells is a critical regulatory component. M2-type macrophages release IL-10 and TGF-β, creating a microenvironment conducive to myogenesis (105). Meanwhile, angiogenic factors such as VEGF promote revascularization of the injured zone, ensuring oxygen and nutrient delivery necessary for new fiber maturation (106). Nutritional status is equally critical, as regeneration is energy-intensive and depends on mitochondrial biogenesis and amino-acid availability (7). Inadequate protein or antioxidant intake slows satellite-cell proliferation and extends recovery time (107). Satellite-cell dynamics also differ between injury types. Repeated microtrauma can deplete the satellite-cell pool, impairing regenerative capacity over time (108). In aging individuals, satellite-cell responsiveness declines, partly due to altered Notch and Wnt signaling and low systemic growth-factor levels (109). Accordingly, interventions that preserve satellite-cell function, including nutritional, pharmacological, and exercise-based strategies, are essential for maintaining musculoskeletal resilience.

### 4.6 Cancer-associated sarcopenia, anabolic resistance, and androgen-regulated muscle resilience

Cancer-associated sarcopenia and cachexia represent clinically important manifestations of the injury–recovery–musculoskeletal resilience axis. Unlike acute sports injury, where tissue damage is often initiated by mechanical overload, cancer-related muscle deterioration is driven by a persistent systemic milieu involving tumor-derived inflammatory mediators, treatment toxicity, endocrine disruption, reduced energy intake, mitochondrial dysfunction, and impaired anabolic signaling (31, 32). These factors interact to produce a chronic injury-like state in which skeletal muscle progressively loses mass, contractile quality, regenerative capacity, and metabolic flexibility (29, 30). A central mechanism in this process is anabolic resistance. Under physiological conditions, amino acids, insulin, IGF-1, mechanical loading, and androgen signaling converge on the PI3K/Akt/mTOR pathway to stimulate protein synthesis and suppress excessive proteolysis (37, 38). In aging and cancer, however, this anabolic responsiveness is blunted. Chronic activation of inflammatory pathways, particularly NF-κB and cytokine-mediated signaling, interferes with insulin/IGF-1 activity, increases ubiquitin–proteasome and autophagy-related degradation, and impairs mTOR-dependent translational control (30, 31, 37). As a result, even when nutritional substrates are available, skeletal muscle may fail to mount an adequate protein-synthetic response. Effective cancer rehabilitation therefore requires an integrated approach that goes beyond protein or amino acid provision by reducing inflammatory interference, improving mitochondrial function, and restoring sensitivity to anabolic stimuli (31, 33, 34).

The interaction between anabolic resistance and aging-associated signaling is also shaped by satellite-cell exhaustion and impaired regenerative competence. In young or metabolically healthy muscle, satellite cells respond to mechanical and biochemical stimuli by proliferating, differentiating, and fusing with damaged fibers. Aging, chronic inflammation, cancer cachexia, and treatment-related toxicity progressively weaken this regenerative reserve. Persistent NF-κB activation, mitochondrial ROS accumulation, altered Notch/Wnt signaling, impaired androgen responsiveness, and reduced IGF-1/Akt/mTOR activity can shift satellite cells toward dysfunction, senescence, or ineffective differentiation. Consequently, muscle repair becomes incomplete even when nutritional substrates and rehabilitation stimuli are provided. Accordingly, successful cancer rehabilitation must address not only muscle protein synthesis but also the upstream inflammatory, endocrine, mitochondrial, and stem-cell regulatory environment that determines whether exercise and nutrition can produce an anabolic response (31, 32, 95, 110).

Mitochondrial dysfunction further amplifies cancer-associated muscle loss. Tumor progression and anticancer therapies can impair electron transport chain activity, reduce ATP generation, promote mitochondrial ROS production, and disturb mitochondrial dynamics and mitophagy (34). When mitochondrial homeostasis becomes insufficient, damaged mitochondria accumulate and reinforce oxidative stress, inflammatory signaling, and activation of cell-death pathways (31, 32, 40). This creates a feed-forward loop in which mitochondrial stress promotes muscle atrophy, while reduced muscle activity and anabolic resistance further compromise mitochondrial renewal. Exercise is therefore particularly relevant because it can stimulate AMPK, PGC-1α, mitochondrial biogenesis, mitophagy coordination, and oxidative metabolism (33, 34). Polyphenols may complement these effects by modulating redox-sensitive pathways such as Nrf2, NF-κB, SIRT1, and AMPK, although their effects should be interpreted as context-dependent signaling modulation rather than simple antioxidant scavenging (111).

Endocrine alterations, especially disruption of androgen signaling, add another important layer to cancer-associated sarcopenia. Androgens regulate skeletal muscle mass and function through the androgen receptor, which promotes myogenic differentiation, satellite cell activity, protein accretion, and muscle fiber hypertrophy (38). Recent findings show that androgen receptor expression is higher in younger skeletal muscle than in older muscle and that androgen receptor signaling can protect skeletal muscle cells against oxidative stress-induced senescence. Mechanistically, androgen stimulation promotes assembly of an androgen receptor/filamin A complex and activates rapid non-genomic signaling through Rac1, focal adhesion kinase, and MAPK/ERK pathways. Disruption of this complex restores the senescent phenotype in C2C12 myoblasts, supporting the idea that androgen receptor-associated signaling contributes to muscle-cell trophism and resistance to oxidative injury (39). This senescence-related mechanism is relevant to cancer rehabilitation because treatment-induced endocrine disruption, chronic inflammation, and mitochondrial ROS accumulation can converge on premature muscle-cell aging, thereby reducing regenerative competence and limiting the functional response to exercise or nutritional support.

This mechanism is particularly relevant in oncology. In prostate cancer, androgen deprivation therapy is a major example of treatment-induced endocrine disruption and is associated with loss of lean mass, increased adiposity, insulin resistance, frailty, and reduced physical function (112). More broadly, cancer cachexia is characterized by an imbalance between catabolic and anabolic processes, frequently accompanied by inflammation, reduced androgen availability, mitochondrial stress, and impaired muscle regeneration (113). Tumor-derived IL-6 and other inflammatory mediators can suppress muscle protein synthesis and accelerate muscle wasting, while chronic systemic inflammation reduces the effectiveness of anabolic signals (114). Therefore, androgen signaling, IGF-1/Akt/mTOR activity, mitochondrial homeostasis, and immune-inflammatory regulation should be considered interconnected determinants of muscle preservation in cancer patients (115).

Skeletal muscle aging studies also indicate that sarcopenia involves nuclear and epigenetic alterations, including chromatin dysregulation, impaired transcriptional activity, altered nuclear morphology, reduced RNA processing, satellite cell dysfunction, and disturbed proteostasis. These nuclear alterations provide an additional mechanistic bridge between aging, chronic inflammation, cancer-associated sarcopenia, and impaired rehabilitation (38, 40). In this context, musculoskeletal resilience depends not only on preventing oxidative damage but also on maintaining transcriptional competence, mitochondrial-nuclear communication, satellite cell function, and endocrine responsiveness (38, 39). Exercise and polyphenol-rich strategies may therefore support cancer rehabilitation by acting at multiple biological levels: reducing chronic inflammatory pressure, improving mitochondrial homeostasis, preserving redox-sensitive adaptive signaling, supporting anabolic responsiveness, and limiting senescence-associated deterioration.

Thus, cancer-associated sarcopenia should be viewed as a multi-system disorder rather than a simple consequence of inactivity or inadequate nutrition. The integration of exercise and polyphenol-based nutritional strategies is mechanistically plausible because these interventions target overlapping nodes of the cachexia–sarcopenia network, including NF-κB-driven inflammation, mitochondrial dysfunction, impaired AMPK/PGC-1α signaling, disrupted IGF-1/Akt/mTOR activity, persistent oxidative stress, and regenerative-cell exhaustion. However, their effectiveness is likely to depend on cancer type, treatment modality, sex, age, baseline endocrine status, nutritional state, and disease stage. This reinforces the need for individualized oncology rehabilitation protocols that combine progressive exercise prescription with targeted nutritional and metabolic support.

### 4.7 Tendon, ligament, and connective-tissue adaptation

Tendons and ligaments transmit mechanical forces between muscle and bone, and their pathophysiology differs from that of contractile tissue (116). Due to limited vascularization, their healing is slower and often results in scar tissue with inferior biomechanical properties (117). The pathogenesis of tendon and ligament injury begins with micro-damage to collagen fibrils and proteoglycan disruption due to repetitive strain (118). This leads to disorganization of the ECM, loss of crimp pattern, and accumulation of mucoid material. Tenocytes, the principal resident cells, respond to mechanical distortion by activating integrin-mediated mechanotransduction pathways (FAK–MAPK–NF-κB). These pathways regulate ECM turnover by coordinating matrix metalloproteinases (MMPs) and their inhibitors (TIMPs) (119). Early after injury, inflammation triggers angiogenesis and fibroblast infiltration, producing type III collagen, which is thinner and less aligned than native type I collagen. During remodeling, type III collagen is progressively replaced by type I collagen, restoring tensile strength under appropriate mechanical loading conditions (120). In contrast, excessive loading or unresolved inflammation promotes chronic tendinopathy, characterized by hypercellularity, neovascularization, and degeneration rather than true healing. Ligament injuries follow a similar pattern. The repair process involves three overlapping phases: inflammation (0–7 days), proliferation (5–21 days), and remodeling (weeks to months) (121). During remodeling, collagen cross-linking and fiber alignment determine mechanical recovery (122). Nutritional factors are integral to this process. Vitamin C (as a cofactor for prolyl/lysyl hydroxylation) and amino acids such as glycine and proline are essential for effective collagen synthesis and ECM maturation (123). Recent insights indicate that tenocytes and fibroblasts exhibit stem-like properties, enabling partial self-renewal. Growth factors such as platelet-derived growth factor (PDGF), basic fibroblast growth factor (bFGF), and connective-tissue growth factor (CTGF) regulate these responses (124). Accordingly, integrating controlled mechanical loading with adequate protein intake, omega-3 fatty acids, and antioxidant support may enhance collagen maturation and reduce reinjury risk (125).

### 4.8 Systemic and metabolic modulation in injury

Sports injury extends beyond local tissue damage and elicits systemic metabolic and hormonal responses that influence healing outcomes. The acute-phase response is characterized by elevated cortisol, catecholamines, and inflammatory cytokines, which mobilize energy substrates but suppress anabolic processes when sustained (80, 126). Energy metabolism becomes a limiting factor in recovery. Injured athletes often experience reduced appetite or restricted intake, leading to low energy availability that impairs protein synthesis and immune competence (127). During immobilization, muscle protein breakdown accelerates through activation of the ubiquitin–proteasome and autophagy–lysosome pathways, while insulin sensitivity declines (128). Adequate energy and amino-acid intake are therefore essential to counteract these catabolic responses and maintain anabolic balance (129). At the tissue level, injury promotes a metabolic shift toward glycolysis and mitochondrial stress. Hypoxia triggers HIF-1α activation, stimulating angiogenesis and glycolytic enzyme expression (90). Simultaneously, pro-inflammatory cytokines alter mitochondrial dynamics, increasing ROS output and reducing ATP production. These effects propagate systemic fatigue and prolong recovery time (130, 131).

Neuroendocrine–immune interactions also shape the repair trajectory. Cortisol limits excessive inflammation but inhibits collagen synthesis and immune-cell proliferation when chronically elevated (71). In contrast, anabolic hormones such as testosterone, growth hormone, and IGF-1 promote protein synthesis and tissue regeneration (132). Systemic effects also involve the gut–muscle axis. Stress hormones and medications can alter gut permeability and microbiota composition, affecting nutrient absorption and immune regulation. Probiotics and anti-inflammatory lipids, particularly omega-3 PUFAs, may help restore this balance (133–135). Thus, energy balance, hormonal regulation, and nutritional quality act as central regulators of the injury–repair continuum. Without adequate systemic support, local tissue repair remains suboptimal. Overall, sports injury should be understood as a continuum of interconnected biological processes rather than discrete stages. Mechanical overload initiates structural damage, triggering the release of damage-associated molecular patterns (DAMPs) and cytokines that recruit immune cells and activate local progenitor populations. The subsequent inflammatory phase is essential for debris clearance and regeneration priming, yet its resolution depends on the balance between pro- and anti-inflammatory signaling.

As inflammation resolves, regenerative processes become dominant, involving coordinated activity of satellite cells, fibroblasts, and endothelial cells to restore ECM structure and vascularization. Mechanical loading provides essential cues for fiber alignment, while nutritional status ensures availability of amino acids and micronutrients required for protein synthesis and collagen maturation. Systemic factors further modulate recovery. Catabolic signals initially mobilize energy reserves but may impair repair if sustained, whereas anabolic mediators such as IGF-1 promote tissue regeneration and protein accretion. Recovery outcomes depend on the coordination of local and systemic responses. Disruption of this balance, through nutrient deficiency, persistent oxidative stress, or inappropriate mechanical loading, may lead to chronic pathology such as fibrosis or tendinopathy. Conversely, when inflammation is regulated, oxidative balance maintained, and nutritional and mechanical inputs are appropriately synchronized, tissues undergo adaptive remodeling, resulting in improved structural integrity and resilience. This integrative perspective highlights that injury and recovery are systemic processes linking biomechanics, immunology, endocrinology, and metabolism, requiring coordinated rehabilitation strategies that combine progressive loading with targeted nutritional support.

## 5 Concept of functional food in sports medicine

The modern concept of functional food has expanded beyond the traditional view of nutrition as a source of energy and basic nourishment. In sports medicine, these foods are now recognized as bioactive nutritional tools that not only sustain physiological function but also accelerate post-exercise recovery, mitigate inflammation, and support long-term adaptation to physical training. Functional food are defined as whole or fortified foods that provide health benefits beyond basic nutrition, often through bioactive compounds such as polyphenols, omega-3 fatty acids, probiotics, and plant-derived bioactives (136–138). These compounds interact with molecular pathways involved in inflammation, oxidative stress, muscle repair, and immune regulation, processes central to athletic recovery and performance optimization. Unlike isolated dietary supplements, which deliver single nutrients (e.g., whey protein or creatine), functional food provide synergistic combinations of nutrients, fibers, and phytochemicals within a natural food matrix. This structural complexity enhances nutrient bioavailability and promotes physiological compatibility, reducing the risk of excessive intake or metabolic imbalance. For example, dairy products such as milk and yogurt provide a balanced mixture of rapidly and slowly digested proteins (whey and casein), along with calcium and electrolytes, creating favorable conditions for muscle protein synthesis, rehydration, and bone health (139, 140). Similarly, fermented dairy products containing probiotics (e.g., *Lactobacillus* and *Bifidobacterium* species) support gut microbiota stability, reduce exercise-induced inflammation, and improve nutrient absorption, thereby enhancing immune function and gastrointestinal integrity during periods of intense training (141, 142).

Polyphenol-rich functional foods should therefore be interpreted not merely as exogenous antioxidants, but as context-dependent regulators of redox-sensitive signaling. Their biological activity depends on dose, timing, chemical structure, bioavailability, gut microbial metabolism, baseline redox status, treatment exposure, and the concurrent exercise stimulus. Rather than simply neutralizing ROS, many polyphenols influence signaling nodes such as Nrf2, NF-κB, AMPK, SIRT1, MAPK, and mitochondrial quality-control pathways, thereby modulating inflammation, cytoprotection, mitochondrial remodeling, autophagy, and adaptive stress responses (143, 144). This distinction is particularly important in oncology rehabilitation, where chronic oxidative stress caused by tumor burden or treatment toxicity may coexist with transient ROS-dependent signals required for exercise adaptation and tissue repair.

Plant- and fruit-based functional foods have also attracted considerable attention because they regulate redox-sensitive and inflammatory signaling rather than acting only as direct antioxidants. Tart cherry juice, rich in anthocyanins, is one of the most extensively studied examples; its consumption has been shown to reduce oxidative stress, accelerate muscle-function recovery, and alleviate delayed-onset muscle soreness (DOMS) following high-intensity exercise (145). However, these effects should be interpreted as modulation of recovery kinetics and redox-inflammatory balance rather than nonspecific ROS neutralization. Similarly, turmeric, containing the polyphenol curcumin, can modulate pro-inflammatory cytokines through inhibition of NF-κB signaling, thereby facilitating resolution of exercise-induced inflammation (146). Other polyphenol-rich foods, including berries, green tea, and dark chocolate, may influence Nrf2-dependent cytoprotective responses, NF-κB-driven inflammatory signaling, AMPK/SIRT1-related mitochondrial regulation, and MAPK-associated stress responses, indicating pathway-specific actions that depend on compound class, dose, formulation, timing, and physiological context (46, 96, 143). Therefore, their benefits during strenuous activity should be viewed as context-dependent redox modulation rather than simple enhancement of systemic antioxidant capacity. Importantly, these benefits are more consistently observed when polyphenols are consumed within whole-food matrices, where synergistic interactions among bioactive compounds support sustained physiological homeostasis (147). Omega-3 fatty acids, particularly eicosapentaenoic acid (EPA) and docosahexaenoic acid (DHA), represent another key class of functional nutrients in sports medicine. Derived from sources such as fish, walnuts, chia seeds, and flaxseeds, these lipids exert anti-inflammatory effects by downregulating prostaglandin synthesis and pro-inflammatory cytokine production (148, 149). Regular intake of omega-3 fatty acids has been associated with reduced muscle soreness, enhanced protein synthesis, and improved endothelial function, benefits particularly relevant for endurance athletes and aging populations. In addition, omega-3 fatty acids modulate gene expression related to mitochondrial biogenesis and lipid metabolism, suggesting a broader role in energy efficiency and muscular adaptation (150–152). The integration of functional food into recovery strategies reflects a shift from isolated supplementation toward comprehensive dietary approaches that emphasize synergistic nutrient interactions. This approach aligns with evidence indicating that food-based interventions more effectively regulate inflammatory and metabolic responses than single-nutrient supplementation (153, 154). Moreover, functional-food–based dietary patterns support long-term health beyond performance by reducing the risk of chronic conditions such as metabolic syndrome, cardiovascular disease, and osteoporosis, which may arise from repeated metabolic stress associated with intensive training (149, 155). Within this framework, functional food can be viewed as multi-target nutritional modulators that bridge clinical nutrition and performance science. They provide immediate benefits, including reduced inflammation and improved recovery kinetics, while also contributing to long-term physiological resilience (156). Thus, diets enriched with functional food represent a holistic strategy for enhancing recovery, improving resistance to oxidative and mechanical stress, and supporting sustained metabolic and musculoskeletal health.

Beyond intracellular redox and inflammatory pathways, receptor-mediated signaling represents another important mechanism through which polyphenols and other nutraceuticals may influence cancer-related tissue responses, particularly by modifying growth-factor responsiveness, proliferation, inflammation, and tissue remodeling. Among these receptors, epidermal growth factor receptor (EGFR/ErbB1/HER1) is particularly relevant because it links extracellular growth factor availability to intracellular cascades involved in proliferation, survival, migration, inflammation, epithelial–mesenchymal transition, angiogenesis, and tissue remodeling (157, 158). Following ligand binding, EGFR undergoes dimerization and tyrosine autophosphorylation, thereby activating downstream RAS/RAF/MEK/ERK, PI3K/Akt/mTOR, JAK/STAT, and PLCγ/PKC pathways. Under physiological conditions, these cascades support controlled tissue repair and remodeling; however, in cancer, EGFR overexpression, mutation, impaired internalization, defective ubiquitination, or crosstalk with other receptor tyrosine kinases can produce sustained proliferative and inflammatory signaling (157, 159). This receptor-centered perspective is relevant to the injury–recovery–musculoskeletal resilience axis because the response to cancer, exercise, and nutritional interventions is not governed only by oxidative stress or inflammation. Growth factor responsiveness and receptor-mediated remodeling also shape whether tissues move toward repair or pathological adaptation. EGFR signaling intersects with MAPK/ERK, PI3K/Akt/mTOR, NF-κB, STAT3, COX-2, VEGF, and matrix remodeling pathways. In cancer-related settings, persistent EGFR activation may reinforce tumor cell survival, cytokine production, angiogenesis, immune evasion, EMT, and stromal remodeling, thereby contributing to an inflammatory and catabolic tissue microenvironment. Conversely, appropriate attenuation of EGFR signaling may help limit pathological proliferation and inflammation while preserving the physiological role of growth factor signaling in repair processes (157, 158).

Emerging evidence indicates that phenolic compounds and agri-food-derived bioactives can modulate EGFR-related signaling through several mechanisms. Some compounds may reduce EGFR tyrosine kinase activity or receptor autophosphorylation, while others may interfere with membrane organization, lipid raft stability, receptor diffusion, or EGFR dimerization. Additional mechanisms include promotion of EGFR ubiquitination, internalization, or degradation, as well as suppression of downstream effectors such as Akt, ERK, STAT3, AP-1, NF-κB, and cyclin D1. For example, curcumin has been reported to reduce EGFR autophosphorylation and downstream Akt/ERK signaling; EGCG may interfere with lipid raft-dependent EGFR dimerization and receptor phosphorylation; quercetin can inhibit EGF-induced EGFR activation and downstream PI3K/Akt signaling; and luteolin has been described as an inhibitor of EGFR-dependent protein kinase activity. These examples further support the view that polyphenols act as multi-target signaling modulators rather than simple ROS scavengers (157, 160–163).

The translational relevance of EGFR modulation is particularly evident in non-small cell lung cancer, where activating EGFR mutations drive tumor survival and EGFR tyrosine kinase inhibitors provide major therapeutic benefit. However, acquired resistance can emerge through secondary EGFR mutations, altered receptor trafficking, sustained receptor retention at the membrane, activation of bypass pathways such as MET or AXL, reactivation of MAPK and PI3K/Akt/mTOR signaling, EMT, autophagy, and immune escape (159). Natural compounds have therefore been investigated as potential partners in combination strategies designed to suppress bypass signaling or resensitize resistant cells. Nevertheless, this field should be interpreted cautiously. Many nutraceuticals show promising EGFR-related activity *in vitro*, but their clinical translation is limited by poor aqueous solubility, low oral bioavailability, rapid metabolism, uncertain tissue exposure, and possible off-target effects (51, 159, 164–166). Therefore, EGFR-related nutraceutical modulation should be framed as a promising mechanistic and translational direction, rather than as an established clinical therapy.

## 6 Functional food in specific sports injury contexts

**Figure 2:**
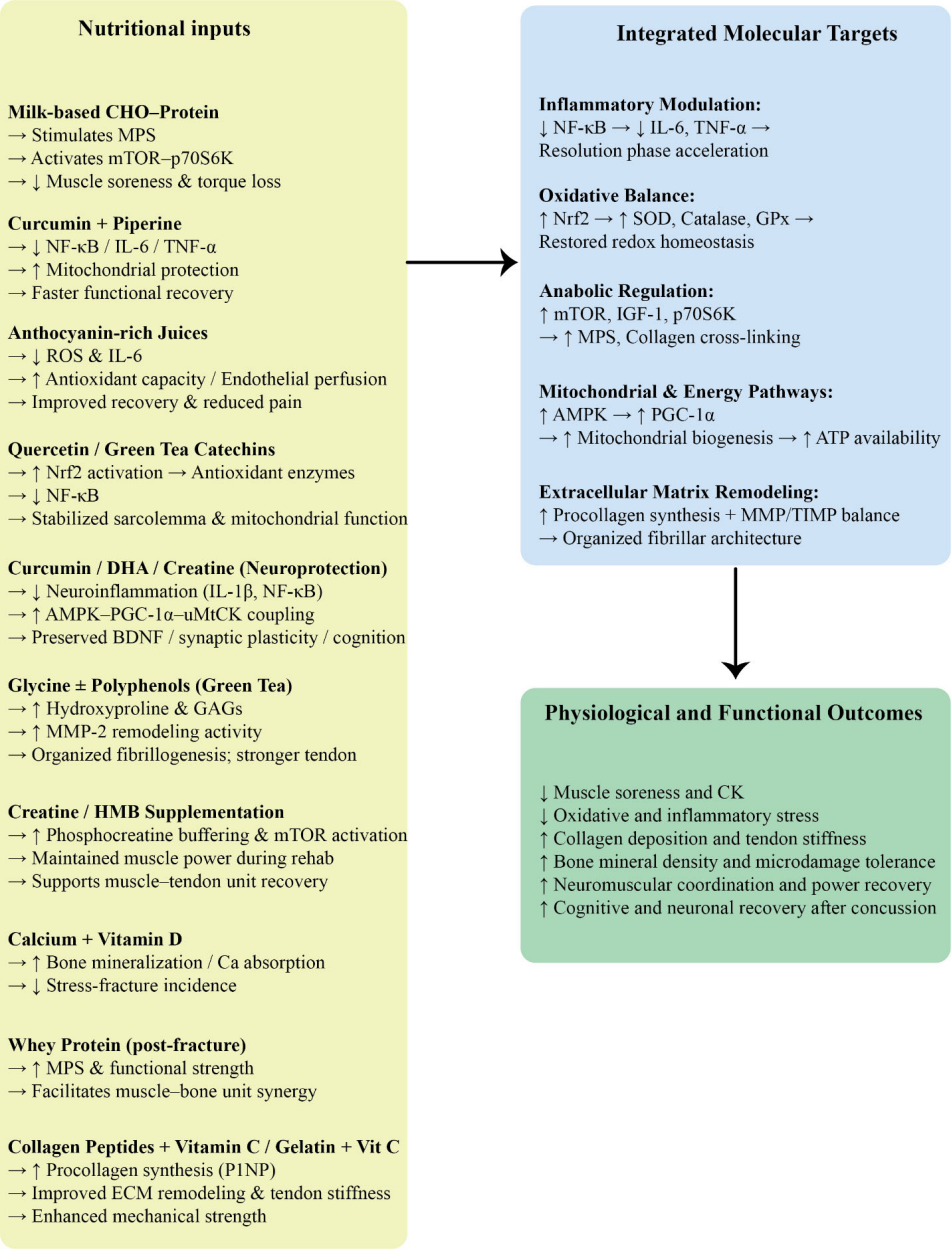
Integrative effects of functional-food–derived nutrients on sports injury recovery and tissue remodeling.

A broad spectrum of experimental and clinical studies has investigated the effects of functional foods and bioactive nutrients across diverse injury models, including exercise-induced muscle damage, tendon and ligament rehabilitation, bone stress injury, and post-concussion recovery. As summarized in **Table 1**, functional-food and bioactive nutritional interventions have been evaluated across diverse sports-injury and recovery contexts, including exercise-induced muscle damage, connective-tissue remodeling, bone stress injury, postoperative rehabilitation, and neurological injury models. Collectively, these studies show that nutritional strategies differ not only in composition, but also in the biological phase of recovery that they are most likely to support. Protein- and carbohydrate-based interventions primarily target early muscle repair and anabolic recovery, collagen- and vitamin C-based strategies are more closely aligned with connective-tissue remodeling, whereas polyphenol-, creatine-, probiotic-, and micronutrient-based approaches appear to act through broader modulation of inflammation, redox balance, neuromuscular function, or systemic recovery capacity. This evidence supports a context-specific view of functional foods, in which clinical relevance depends on matching the intervention to the injured tissue, recovery phase, physiological status, and rehabilitation goal. **Figure 2** schematically illustrates how redox regulation, inflammatory control, mitochondrial adaptation, and anabolic signaling interact during tissue repair and functional recovery, while the mechanistic details and specific contexts of these interventions are discussed in the following subsections.

**Table 1:** Functional-food and bioactive nutritional interventions in sports-injury and recovery contexts.

Experimental model/population	Intervention/dose	Key findings	Prioritized mechanistic module/biological targets	Translational relevance/clinical implication	Ref.
32 healthy males; four groups	Milk-based CHO–protein supplement before, immediately after, or 24 h post-exercise	Post- and 24-h groups had less muscle soreness and torque loss	Timing-dependent stimulation of muscle protein synthesis; whey/casein synergy; mTOR–p70S6K activation	Supports early post-exercise or post-injury nutrient timing as a practical strategy to accelerate muscle recovery and preserve contractile function	(167)
10 elite rugby players; cross-over design	Curcumin + piperine supplementation before and after EIMD	Improved sprint recovery and reduced CK at 72 h	Anti-inflammatory and redox modulation; NF-κB suppression; improved mitochondrial protection; piperine-enhanced curcumin exposure	Relevant for high-intensity team-sport recovery, especially when inflammation and muscle membrane disruption dominate the recovery profile	(168)
12 trained men	Quercetin 1000 mg/day for 14 days before eccentric exercise	Attenuated loss of isometric strength and lower conduction-velocity decay	Stabilized sarcolemmal potential; reduced oxidative stress; preserved muscle fiber conduction velocity	Suggests potential benefit for neuromuscular preservation after eccentric stress, although response may depend on training status and baseline oxidative stress	(169)
30 healthy adults	Quercetin 1000 mg/day for 7 days before and 5 days after eccentric contractions	No significant effect on soreness or CK, but maintained plasma quercetin	Variable response related to bioavailability; possible ceiling effect in mild injury	Highlights that quercetin efficacy is not universal and may require stronger injury stimulus, optimized formulation, or responder-based stratification	(170)
20 active females	Tart Montmorency cherry juice, 30 mL twice daily for 8 days	Faster jump recovery; trend toward lower soreness	Anthocyanin-driven modulation of oxidative stress; IL-6 regulation; enhanced vascular perfusion	Practical whole-food intervention for recovery kinetics, particularly when soreness and explosive-performance recovery are relevant outcomes	(171)
14 male students; cross-over design	Tart cherry juice, 12 oz twice daily for 8 days	Strength loss decreased from 22% in placebo to 4%; lower pain	Antioxidant and anti-inflammatory polyphenols reduce excessive ROS and myofibrillar damage	Supports tart cherry juice as a clinically feasible strategy for reducing functional loss after damaging exercise	(172)
16 students	Black currant nectar, 16 oz twice daily for 8 days	Reduced CK and IL-6; improved antioxidant capacity	Anthocyanins reduce NF-κB activation and lipid peroxidation	Indicates potential value of anthocyanin-rich beverages for biochemical recovery, although functional outcome validation remains needed	(173)
20 recreational runners; half-marathon model	New Zealand blackcurrant extract, 2 × 300 mg/day for 7 days before and 2 days after exercise	No significant improvement in soreness or performance	Inadequate dose–response; IL-6 increased in supplement group	Demonstrates exercise-context dependency; effects may differ between eccentric injury models and endurance-race recovery	(174)
36 non-resistance-trained men	Tart cherry vs pomegranate vs placebo	No significant group difference	Possible population effect; mild eccentric stress may have been insufficient to elicit protective effects	Reinforces the need to match polyphenol interventions to injury severity, baseline training status, and measurable recovery endpoints	(175)
30 healthy men	Anthocyanin-rich antioxidant juice, 240 mL twice daily for 9 days	Faster recovery of running economy; lower soreness and CK	Polyphenols attenuate oxidative damage; improved mitochondrial efficiency	Suggests translational relevance for endurance recovery and repeated training when running economy is a key functional outcome	(176)
20 untrained men	Green tea extract, 500 mg/day for 15 days	Reduced CK and muscle-damage markers; soreness unchanged	Catechins stabilize redox state and reduce membrane leakage	Supports biochemical protection, but limited effect on subjective recovery suggests that molecular and functional outcomes should be interpreted separately	(177)
50 athletes with chronic ankle instability	Specific collagen peptides, 5 g/day vs placebo for 6 months	Greater improvements in CAIT and FAAM-G scores; fewer re-injuries at 3-month follow-up	Collagen peptide substrate supply for ECM remodeling; symptom and function gains despite no arthrometer change suggest neuromuscular/ECM remodeling support	High translational relevance for recurrent ankle injury and chronic instability, especially as an adjunct to rehabilitation	(178)
147 student athletes; 97 analyzed	Collagen hydrolysate, 10 g/day vs placebo for 24 weeks	Reduced activity-related joint pain across multiple tasks; stronger effect in knee-arthralgia subgroup	Augmented cartilage/ECM turnover; analgesic effect likely through improved collagen matrix and reduced inflammatory signaling	Relevant for athletes with activity-related joint pain, but effects may be strongest in symptomatic subgroups	(179)
8 healthy males; cross-over engineered ligament model	Vitamin C-enriched gelatin, 5 or 15 g, taken 1 h before skipping, 3 times/day for 3 days	15 g + vitamin C doubled circulating P1NP; serum-treated ligaments showed increased collagen content and mechanics	Rapid rise in glycine, proline, and hydroxyproline; vitamin C as cofactor for prolyl/lysyl hydroxylases; increased collagen synthesis capacity	Strong mechanistic support for timing collagen/gelatin intake before loading to enhance connective-tissue remodeling	(180)
10 recreational males; cross-over design	15 g vitamin C-enriched gelatin, hydrolyzed collagen, or gummy, 1 h before jump rope	Gelatin and hydrolyzed collagen tended to increase P1NP by about 20%; high interindividual variability	Similar amino acid availability across forms; timing with brief loading likely key driver of collagen anabolism	Supports collagen-targeted nutrition before mechanical loading, but variability indicates need for individualized or repeated-measure protocols	(181)
2 elite rugby athletes after ACL reconstruction; case report	Structured rehabilitation + gelatin 15 g + vitamin C timed before collagen-focused loading	Return to baseline body composition by about 24 weeks; strength and knee function restored by about 30 weeks	Targeted collagen nutrition + mechanotherapy supports graft/ligament and periarticular connective-tissue remodeling	Clinically illustrative but low-level evidence; useful for hypothesis generation in ACL rehabilitation protocols	(182)
18 adolescent fin swimmers with tendon overuse	Creatine supplementation vs placebo during conservative rehabilitation	Faster gains in plantarflexion peak torque; quicker pain reduction; lower CK increase	Elevated phosphocreatine buffering supports training load during rehabilitation; indirect facilitation of tendon remodeling	Suggests creatine may support rehabilitation capacity and muscle performance in tendon overuse contexts, although tendon-specific mechanisms require further validation	(183)
Rat Achilles tendinitis model	Green tea catechins ± glycine diet for 7 or 21 days	Better collagen bundle organization; higher hydroxyproline; increased MMP-2; greater load to failure with green tea + glycine	Glycine as major collagen amino acid + catechins promote ECM synthesis, remodeling, and biomechanical strength	Strong preclinical support for combined collagen-substrate and polyphenol strategies in tendon remodeling; human validation needed	(184)
Rat Achilles inflammation model	5% dietary glycine for 8 or 22 days	Increased hydroxyproline and GAGs; increased MMP-2; higher birefringence; stronger tendons at rupture	Glycine accelerates collagen reconstitution and organized fibrillogenesis; enhanced tendon resistance	Supports glycine availability as a mechanistic determinant of tendon repair, but dose translation to humans remains uncertain	(185)
8 athletes with patellar tendinopathy	4-week eccentric + stretching + ESWT ± HMB	Both groups improved strength; HMB group showed added gains in power metrics	HMB may support muscle power during tendon rehabilitation through leucine-metabolite effects on mTOR signaling; tendon pain unchanged	May improve performance-related outputs during tendon rehabilitation, but tendon-specific benefit remains unclear	(186)
30 male athletes after ACL reconstruction	Glucosamine sulfate 1000 mg/day for 8 weeks vs placebo + same rehabilitation	Pain and function improved in both groups; no between-group differences	Glucosamine not additive in this protocol; cartilage-centric effects may not translate in early ACL rehabilitation window	Indicates that symptom improvement may be driven primarily by rehabilitation; glucosamine should not be assumed beneficial in early ACL recovery	(187)
5201 female Navy recruits during 8-week basic training	Calcium 2000 mg/day + vitamin D 800 IU/day vs placebo	About 20–21% lower stress-fracture incidence in supplemented group	Improved calcium balance and bone turnover; vitamin D-mediated calcium absorption; improved microdamage tolerance under repetitive load	High translational relevance for stress-fracture prevention in high-load female populations, especially when calcium/vitamin D insufficiency risk is present	(188)
133 elite rowers; cross-sectional study	Observational study of diet restriction, vitamin D/K status, calcium intake, BMD, and rib stress injury history	Rib stress injury linked with lower rib/spine/femur BMD; diet restriction and menstrual dysfunction associated with lower BMD; sex/weight-class effects	Energy availability, endocrine status, menstrual function, and mineral/vitamin sufficiency underpin rib BMD and stress-injury risk	Supports screening for low energy availability, menstrual dysfunction, and micronutrient status in athletes at risk of bone stress injury	(189)
38 elderly hip-fracture patients; early postoperative phase	Whey protein 32.2 g before and after rehabilitation session for 2 weeks vs control	Greater improvements in knee-extension strength in operated limb; better Barthel Index transfer/walking/toilet-use scores	High-leucine whey augments muscle protein synthesis during rehabilitation; supports muscle–bone unit recovery after surgery	Clinically relevant to postoperative rehabilitation and frailty-related muscle weakness, although not a sports-injury model	(190)
20 indoor elite wheelchair athletes with vitamin D insufficiency	Vitamin D 6000 IU/day for 12 weeks; double-blind design	25(OH)D normalized in all; no change in Wingate; minor strength gains in non-dominant arm	Restored vitamin D status supports musculoskeletal health; performance effects context-dependent; bone benefit inferred via status correction	Indicates that correcting deficiency is important for musculoskeletal health, but performance gains may require combined training or longer follow-up	(191)
Rat model of mild fluid-percussion brain injury	Dietary curcumin, 500 ppm for 4 weeks before injury	Prevented decline in hippocampal AMPK, uMtCK, and COX-II; improved cognitive function	Restored mitochondrial energy homeostasis; activated AMPK–PGC-1α axis; upregulated antioxidant enzymes	Preclinical support for curcumin in neuro-metabolic resilience after brain injury; human concussion evidence remains limited	(192)
Mouse controlled-cortical-impact TBI	Curcumin 75–300 mg/kg before and after injury	Reduced brain-water content; improved neurological score	Inhibited IL-1β/NF-κB signaling; reduced aquaporin-4 expression; stabilized blood–brain barrier integrity	Suggests curcumin may reduce edema and neuroinflammation after TBI, but dose translation and clinical safety require validation	(193)
Rat cortical contusion trauma	Curcumin 50–100 mg/kg for 5 days before injury	Reduced lesion volume and MDA; improved motor recovery	Suppressed lipid peroxidation; increased GSH activity; restored neuronal viability	Supports antioxidant and neuroprotective mechanisms in acute brain injury, but remains preclinical	(194)
Human elite rugby players; cross-over RCT	Oral curcumin + piperine before and after EIMD	Reduced CK increase; better sprint power 24 h post-exercise	Enhanced bioavailability through piperine; systemic anti-inflammatory and membrane-protective effects	Practical sports-recovery application, but optimal dose and timing remain to be standardized	(168)
Healthy volunteers and animals	Curcumin 2 g ± piperine 20 mg	Piperine increased curcumin bioavailability by about 2000%	Inhibition of hepatic and intestinal glucuronidation pathways leading to greater systemic retention	Important pharmacokinetic evidence explaining why curcumin formulation strongly affects translational efficacy	(195)

### 6.1 Muscle injury and regeneration

Skeletal-muscle injury is among the most common consequences of intensive or unaccustomed exercise and constitutes a central focus in sports nutrition research. Exercise-induced muscle damage (EIMD) leads to transient decrements in contractile performance, delayed-onset muscle soreness (DOMS), membrane leakage of intracellular enzymes such as creatine kinase (CK) and lactate dehydrogenase (LDH), and activation of inflammatory and oxidative pathways. Functional foods enriched with bioactive nutrients have emerged as recovery-oriented strategies that modulate oxidative stress, inflammation, and mitochondrial function. Adequate amino-acid availability is fundamental for post-injury anabolism. Cockburn, Stevenson (167) demonstrated that the timing of milk-based carbohydrate–protein (CHO-P) ingestion critically determines recovery kinetics. The groups ingesting milk immediately or 24 h after exercise exhibited smaller reductions in torque and soreness than the pre-exercise group, confirming that synchronized nutrient delivery during the early repair window sustains muscle-protein synthesis and suppresses proteolysis. The dual presence of rapidly digested whey and slowly digested casein provides both an acute leucine spike to activate mTOR and prolonged amino-acid availability to maintain translational signaling (196, 197).

Beyond dairy, leucine-rich formulations and β-hydroxy-β-methyl-butyrate (HMB) fortification attenuate ubiquitin–proteasome-mediated breakdown and enhance p70S6K phosphorylation (197, 198). However, whole-food matrices appear more effective than isolated amino-acid powders because accompanying carbohydrates augment insulin release and intracellular amino-acid uptake (196, 199). Collectively, these findings highlight that functional recovery food combining protein and carbohydrate create a favorable anabolic milieu that isolated supplements rarely reproduce. Among plant-derived compounds, curcumin, the principal polyphenol in turmeric, has attracted considerable attention for its anti-inflammatory potential in EIMD. Delecroix, Abaïdia (168) investigated curcumin combined with piperine in elite rugby players. The curcumin–piperine condition displayed a markedly smaller reduction in sprint mean power compared with placebo, demonstrating partial preservation of contractile performance. Mechanistically, curcumin down-regulates NF-κB and COX-2 signaling, thereby reducing IL-6 and TNF-α production, while piperine enhances systemic availability by inhibiting glucuronidation, prolonging its biological activity (195, 200). Experimental models also show that curcumin stabilizes mitochondrial membranes, maintains ATP production, and limits calcium-dependent protease activation (201, 202).

Functional food rich in anthocyanins demonstrate reproducible benefits for EIMD recovery. Connolly, McHugh and Padilla-Zakour (172) reported substantial reductions in strength loss and soreness following tart-cherry juice consumption. These protective effects were attributed to anthocyanin-mediated attenuation of oxidative stress and inflammatory signaling. Subsequent studies confirmed improvements in recovery kinetics, although variability in outcomes has been observed depending on dose, exercise model, and population characteristics (171, 173, 174, 176). Overall, anthocyanins appear to accelerate recovery primarily through suppression of ROS generation, modulation of cytokine cascades, and improvement of mitochondrial and vascular function (203, 204). Quercetin exhibits redox-modulatory and mitochondrial-protective properties. Bazzucchi, Patrizio (169) demonstrated preservation of muscle strength and reduced neuromuscular impairment following supplementation. However, other studies reported limited effects on biochemical markers, likely reflecting variability in bioavailability and supplementation form (170, 205). Mechanistically, quercetin regulates redox balance through activation of Nrf2 and suppression of NF-κB signaling, thereby maintaining mitochondrial function and cellular integrity (206). Whole-food sources therefore likely yield more consistent physiological outcomes than isolated quercetin capsules.

Polyphenols from green tea, particularly epigallocatechin gallate (EGCG), provide additional redox-modulatory support. da Silva, Machado (177) demonstrated reductions in CK and LDH following supplementation. Catechins modulate lipid peroxidation and enhance endogenous antioxidant defenses, although subjective recovery outcomes may not always parallel biochemical improvements (207). EGCG also promotes Nrf2 activation and up-regulation of endogenous antioxidant enzymes such as SOD, catalase, glutathione peroxidase, while modulating MAPK signaling to favor cell survival (177). Regular consumption of green-tea beverages or catechin-fortified snacks may therefore confer moderate but sustained cytoprotective effects during repeated training cycles. Across these interventions, the key issue is not whether each nutrient targets a separate pathway, but how nutritional and exercise-derived signals reshape the injury microenvironment as an integrated and context-dependent network. Eccentric exercise initially induces sarcolemmal disruption, calcium influx, mitochondrial ROS production, cytokine release, and transient impairment of contractile function. When this response remains controlled, ROS and inflammatory mediators contribute to adaptive remodeling; however, when it becomes prolonged or excessive, the same signals reinforce NF-κB activation, mitochondrial dysfunction, proteolysis, and impaired regeneration. In this context, polyphenols, omega-3 fatty acids, and protein-rich functional foods should be viewed as complementary modulators of the transition from damage signaling to repair signaling. Polyphenols may limit excessive NF-κB-driven inflammation while preserving adaptive Nrf2-related redox responses; omega-3 fatty acids may shift lipid mediator balance toward resolution; and protein–carbohydrate matrices may restore amino acid availability and insulin-supported mTOR responsiveness. Together, these actions converge on a common biological objective: reducing maladaptive inflammatory and oxidative amplification while preserving the anabolic, mitochondrial, and redox signals required for functional recovery (208–211). Importantly, these effects should be interpreted through a hormetic and context-dependent redox framework. During exercise, a moderate increase in ROS is not necessarily pathological; rather, it can serve as a signal for mitochondrial renewal, antioxidant enzyme induction, angiogenic adaptation, and metabolic remodeling. Therefore, polyphenols may be most beneficial when they attenuate excessive or prolonged oxidative-inflammatory stress without abolishing transient redox signals needed for adaptation. Inconsistencies across studies are better interpreted as consequences of biological and methodological context rather than as direct contradictions. Improvements in soreness, biochemical recovery, or inflammatory balance are more likely when polyphenol intake is matched to a state of elevated oxidative-inflammatory stress, whereas effects on performance or training adaptation may be modest when the redox stimulus is low, the dose is insufficient, or the intervention is poorly timed. Training status, baseline oxidative stress, food matrix, gut-derived metabolites, dose, timing relative to exercise, and intervention duration can all shift the response toward either enhanced recovery or potential interference with adaptive signaling (98, 212). Despite generally positive outcomes, not all studies report consistent benefits of polyphenol supplementation on muscle recovery. For instance, while anthocyanin-rich interventions frequently reduce soreness and oxidative markers (171–173), yet some studies show minimal improvement in functional recovery following endurance exercise (174). Similarly, quercetin supplementation demonstrates protective effects in some models (169), whereas others report no significant changes in inflammatory or muscle-damage biomarkers (205). These discrepancies reinforce the context-dependent nature of polyphenol efficacy and highlight the need to optimize delivery form, dosage, timing, and interaction with exercise-induced metabolic stress. Collectively, functional foods should be viewed as modulators rather than suppressors of the injury response, preserving essential adaptive signaling while limiting excessive damage. Their effectiveness arises from the integrated regulation of inflammatory, redox, mitochondrial, and anabolic processes that underpin muscle regeneration.

### 6.2 Tendon and ligament healing

Tendon and ligament injuries are slow to repair because these tissues are relatively hypocellular and hypovascular, making substrate delivery and matrix remodeling rate-limiting.

Accordingly, nutritional strategies that enhance collagen synthesis, regulate the matrix metalloproteinase (MMP) milieu, and support load-responsive remodeling can complement rehabilitation and improve recovery quality. Collagen-rich functional food provides glycine, proline, and hydroxyproline, the core amino acids required for collagen triple-helix formation and cross-linking. In a randomized, double-blind crossover study, ingestion of 15 g vitamin C–enriched gelatin one hour before brief skipping bouts doubled circulating procollagen type I N-terminal peptide, indicating an acute acceleration of collagen synthesis when nutrient availability coincides with tendon loading (180). Follow-up work demonstrated that both hydrolyzed collagen and vitamin C–enriched gelatin administered ~60 min before exercise elevate circulating amino acids and increase procollagen markers, highlighting the importance of timing rather than collagen format (181). In applied rehabilitation, a case report in elite rugby athletes integrating gelatin plus vitamin C within a structured ACL program reported preserved body composition, earlier restoration of leg strength, and return to baseline knee function by ~30 weeks, supporting feasibility and translational relevance (182). Longer supplementation periods may further relieve joint discomfort: a 24-week randomized, placebo-controlled trial in competitive athletes showed significant reductions in activity-related joint pain with collagen hydrolysate, particularly in those with knee arthralgia (179). Similarly, six months of specific collagen peptides (5 g/day) improved subjective ankle stability and reduced reinjury rates, despite minimal changes in instrumented stiffness, suggesting early functional benefits preceding structural adaptation (178). Mechanistically, these effects are consistent with nutrient-timed delivery of collagen substrates and vitamin C, which supports prolyl/lysyl hydroxylation, enhances fibrillogenesis, and improves tensile properties when combined with mechanical loading.

Glycine, with or without green tea polyphenols, has also been investigated as a strategy to support tendon remodeling following inflammation. Glycine is the most abundant amino acid in collagen and may modulate inflammatory cascades relevant to tendinopathy. In rat Achilles tendinitis, a 5% glycine diet increased hydroxyproline and glycosaminoglycan content, improved collagen organization, and enhanced load-to-failure during remodeling, indicating faster and stronger tissue reconstitution (185). When combined with green tea extract, tendons exhibited greater hydroxyproline levels, increased MMP-2 activity, improved collagen organization, and superior mechanical resistance, suggesting additive effects from combining anti-inflammatory polyphenols with a collagen-focused amino acid profile (184). These findings support a strategy of combining glycine-rich proteins with polyphenols to optimize matrix turnover and fiber alignment.

Although creatine is primarily associated with muscle metabolism, it may also support tendon rehabilitation. A randomized, double-blind study in adolescent fin swimmers with tendon overuse injury showed that creatine supplementation preserved lean mass during immobilization, enhanced gains in plantar-flexion peak torque, reduced pain scores, and attenuated creatine kinase elevations (183). These findings suggest improved tissue tolerance to loading, potentially through enhanced local energy buffering and improved rehabilitation capacity. In a pilot double-blind trial, β-hydroxy-β-methylbutyrate (HMB) supplementation enhanced concentric power during a 4-week rehabilitation program without affecting lean mass or pain, indicating early neuromuscular benefits that may support progression of tendon-specific loading (186). Collectively, tendon and ligament healing appears to benefit most when nutrient delivery is synchronized with mechanical loading. Ingesting collagen or gelatin with vitamin C approximately 30–60 minutes before exercise provides substrates and cofactors during periods of increased perfusion and fibroblast activity (180–182). Longer-term supplementation with collagen peptides may further improve pain and perceived function (178, 179), while glycine and polyphenol combinations support matrix remodeling and mechanical resilience (184, 185). Creatine and HMB may enhance the muscle–tendon unit by improving energy availability and neuromuscular performance during rehabilitation (183, 186). Importantly, these nutritional strategies should be integrated within progressive, tendon-specific loading programs, where they amplify mechanotransduction rather than replace mechanical stimuli. Beyond these mechanistic insights, an important consideration is that the majority of evidence supporting collagen-targeted interventions remains either acute (biomarker-based) or derived from small cohorts. While increases in circulating procollagen markers (181, 182) provide strong biochemical evidence of enhanced collagen synthesis, the extent to which these acute responses translate into long-term improvements in tendon mechanical properties remains less clearly established. Similarly, although subjective outcomes such as pain reduction and perceived stability improve with prolonged supplementation (178, 185), objective biomechanical parameters often show smaller or delayed changes, suggesting a temporal disconnect between functional perception and structural remodeling. In addition, variability in rehabilitation protocols introduces further complexity. Differences in loading type (eccentric vs. heavy slow resistance), frequency, and intensity may significantly influence how nutritional interventions interact with mechanotransduction pathways. For example, collagen synthesis is highly sensitive to mechanical strain magnitude and timing, meaning that identical nutritional strategies may yield different outcomes depending on exercise design. Moreover, inter-individual variability in collagen turnover, age, and baseline nutritional status may further modulate responsiveness. Taken together, current evidence supports a synergistic model in which nutritional strategies enhance, but do not replace, load-driven adaptation. However, the heterogeneity of study designs and outcome measures limits direct comparison across studies, emphasizing the need for standardized, long-term human trials integrating biochemical, structural, and functional endpoints.

### 6.3 Bone stress and fracture recovery

Adequate calcium and vitamin D remain the cornerstone of nutritional strategies to maintain bone strength and reduce stress-fracture risk among athletes exposed to repetitive mechanical loading. In a landmark randomized, double-blind, placebo-controlled trial, Lappe, Cullen (188) examined more than 5–000 female Navy recruits during eight weeks of basic training and demonstrated that daily supplementation with 2–000 mg calcium and 800 IU vitamin D reduced stress-fracture incidence by approximately 20%. These findings confirm that optimized mineral availability enhances bone micro-resistance under repetitive mechanical strain. Mechanistically, this combination improves intestinal calcium absorption, suppresses parathyroid hormone (PTH) hypersecretion, and promotes balanced bone turnover, thereby preserving trabecular integrity during repetitive loading. Vitamin D deficiency remains prevalent in both indoor and endurance athletes, potentially compromising bone integrity through reduced calcium absorption, impaired osteoblast function, and altered RANKL/osteoprotegerin (OPG) signaling.

Flueck, Schlaepfer and Perret (191) demonstrated that supplementation with 6–000 IU/day vitamin D for 12 weeks normalized 25(OH)D levels in elite wheelchair athletes with insufficient baseline status. Although functional performance changes were limited, restoring vitamin D sufficiency is essential for skeletal mineralization and muscle–bone crosstalk, emphasizing that correction of deficiency is a prerequisite for both performance and fracture prevention. Beyond single-nutrient effects, bone stress injuries arise from complex interactions among energy availability, endocrine regulation, and micronutrient status. Lundy, Suni (189) assessed 133 elite rowers and found that those with rib-stress injuries exhibited markedly lower bone mineral density (BMD) in the ribs, spine, and femur. Energy restriction and menstrual dysfunction were independently associated with lower BMD and higher injury risk, whereas adequate calcium, vitamin D, and vitamin K intake correlated with improved bone status. These findings highlight that fracture susceptibility is not solely mechanical but strongly influenced by metabolic factors, where low energy availability suppresses the hypothalamic–pituitary–gonadal axis, reduces sex-hormone levels, and downregulates bone-formation pathways such as Wnt/β-catenin and IGF-1 signaling. Accordingly, functional food that support energy balance, particularly dairy and protein-rich matrices providing both calcium and amino acids, may deliver combined anabolic and mineral benefits for bone remodeling under load.

The recovery period following fracture or orthopedic surgery is characterized by immobilization-induced muscle atrophy, reduced mechanical loading, and a transient catabolic state that can delay bone union. In a controlled clinical study, Niitsu, Ichinose (190) showed that supplementation with 32 g whey protein before and after rehabilitation sessions for two weeks significantly improved knee-extension strength and functional mobility in elderly hip-fracture patients. These findings support the concept that high-leucine whey protein enhances muscle anabolism, thereby increasing mechanical loading on bone through the muscle–bone unit. The rapid digestibility and essential-amino-acid profile of whey, particularly leucine, likely activate the mTOR/p70S6K pathway, supporting both muscle and osteoblastic protein synthesis. Emerging evidence further indicates that insufficient protein intake may impair collagen cross-linking and osteoid formation, especially when combined with inadequate calcium intake. Therefore, protein-containing functional food, including fortified dairy, soy-based products, and collagen-peptide formulations, can serve as effective adjuncts in bone rehabilitation when energy and mineral requirements are met.

Despite strong evidence supporting calcium and vitamin D supplementation (188, 191), their isolated effects should be interpreted within the broader context of systemic energy availability and endocrine regulation. In many athlete populations, particularly those experiencing relative energy deficiency, bone health is compromised not only by insufficient mineral intake but also by suppressed anabolic hormones and impaired metabolic signaling. Consequently, interventions targeting single nutrients may yield limited benefits if underlying energy imbalance is not corrected. Furthermore, discrepancies across studies suggest that the magnitude of benefit from nutritional interventions depends on baseline deficiency status. For instance, vitamin D supplementation produces substantial improvements in individuals with insufficient levels (191), whereas its effects are minimal in already sufficient populations. Similarly, protein supplementation enhances functional recovery following fracture (190), yet its direct impact on bone mineral density remains less pronounced when calcium intake is adequate. Another critical limitation lies in the difficulty of isolating nutritional effects from mechanical influences. Bone adaptation is inherently mechanosensitive, and variations in loading patterns during rehabilitation can significantly confound interpretation of nutritional outcomes. This interaction underscores that bone recovery is governed by an integrated mechanobiological system rather than discrete nutritional inputs. Therefore, while functional food strategies clearly contribute to bone resilience and recovery, their effectiveness is contingent upon adequate energy intake, hormonal balance, and appropriate mechanical loading. Future research should adopt multifactorial designs to better delineate the relative contributions of nutrition, endocrine status, and biomechanical stimuli.

### 6.4 Neuroprotection and concussion recovery

Traumatic brain injury (TBI) initiates a complex cascade of biochemical events that extend beyond the initial mechanical insult. The secondary phase is characterized by mitochondrial dysfunction, oxidative stress, ionic imbalance, neuroinflammation, and cytoskeletal disruption, all of which contribute to progressive neuronal damage. Emerging evidence indicates that specific bioactive nutrients, particularly curcumin, omega-3 fatty acids, and creatine, can modulate these processes and support neuroregeneration. The polyphenolic compound curcumin exhibits potent antioxidant and anti-inflammatory properties that target multiple convergent mechanisms of TBI. In a controlled cortical impact model, Laird, Sukumari-Ramesh (193) demonstrated that both pre- and early post-treatment with curcumin significantly reduced cerebral edema and improved neurological outcomes. Mechanistically, curcumin suppressed interleukin-1β (IL-1β) up-regulation and inhibited aquaporin-4 (AQP4) expression in astrocytes, thereby reducing cytotoxic swelling. This effect was mediated through inhibition of NF-κB signaling, resulting in decreased astrocytic activation and GFAP expression. These findings identify curcumin as a modulator of the IL-1β–AQP4 axis, limiting astrocyte-driven edema formation following neurotrauma.

Complementary evidence from Samini, Samarghandian (194) further supports curcumin’s neuroprotective role. Pretreatment with 50–100 mg/kg curcumin for five days reduced lesion volume, suppressed lipid peroxidation (MDA), and improved motor performance, indicating reduced oxidative damage and enhanced functional recovery. Post-traumatic energy crisis, characterized by impaired ATP production and mitochondrial dysfunction, represents a key determinant of neuronal vulnerability. Sharma, Zhuang (192) showed that dietary curcumin (500 ppm for four weeks) restored AMPK, uMtCK, and COX-II levels in the hippocampus following injury, indicating recovery of mitochondrial energy regulation. Wu, Ying and Gomez-Pinilla (213) reported normalization of BDNF, synapsin I, and CREB expression, linking curcumin to improved synaptic plasticity and cognitive recovery. Collectively, these findings demonstrate that curcumin preserves mitochondrial function and neurotrophic signaling, thereby supporting neuronal survival and cognitive function. Omega-3 polyunsaturated fatty acids (PUFAs), particularly DHA, are integral to neuronal membranes and regulate synaptic and metabolic function. Wu, Ying and Gomez-Pinilla (214) demonstrated that four weeks of omega-3 supplementation normalized BDNF, synapsin I, and CREB expression and improved spatial learning following TBI. Subsequent work showed restoration of Sir2α and AMPK signaling and reduction of oxidative damage, indicating improved energy homeostasis (215). Additional studies confirm the multifaceted neuroprotective effects of omega-3 fatty acids. In a repetitive mild-TBI model, Wang, Van (216) reported improved spatial learning and faster physiological recovery in animals fed a fish-oil diet. Similarly, Pu, Guo (217) showed preservation of hippocampal neurons, reduced inflammation, and maintenance of myelin integrity, indicating protection against white-matter damage. The prophylactic potential of DHA was further demonstrated by Mills, Hadley and Bailes (218), where pre-injury supplementation reduced axonal damage, apoptosis, and microglial activation while improving memory performance. Collectively, DHA and EPA act on multiple molecular targets, including inflammatory signaling, mitochondrial regulation, and synaptic maintenance, ultimately improving neuronal resilience and recovery. Creatine is a guanidino compound that facilitates rapid ATP regeneration via the phosphocreatine system and stabilizes cellular energy charge under hypoxic conditions. Scheff and Dhillon (219) demonstrated that dietary creatine (0.5–1% for two weeks) reduced accumulation of lactate and free fatty acids following TBI, indicating attenuation of metabolic stress. The effect was dose-dependent, with higher intake producing greater neuroprotection. By maintaining phosphocreatine reserves, creatine limits energy failure, reduces excitotoxic damage, and supports mitochondrial stability.

Despite extensive mechanistic evidence, the clinical applicability of functional-food strategies depends critically on appropriate dosing, timing, and delivery format. In human studies, polyphenol intake typically ranges between 300–1000 mg/day (e.g., curcumin, quercetin), while omega-3 fatty acids are commonly administered at 1–3 g/day EPA+DHA to achieve anti-inflammatory effects. Anthocyanin-rich interventions, such as tart cherry juice, are generally provided as 240–480 mL/day or ~30 mL concentrate twice daily for 7–10 days, particularly around periods of high-intensity exercise. Timing also plays a decisive role. Protein- and collagen-containing functional foods are most effective when consumed within the early post-exercise window (0–24 h) or 30–60 min prior to mechanical loading, where increased tissue perfusion enhances substrate delivery and anabolic signaling. Similarly, polyphenols appear most beneficial when administered before and during periods of oxidative stress or inflammation, rather than chronically at high doses that may blunt adaptive responses. Importantly, whole-food matrices (e.g., dairy, berries, green tea) provide superior bioavailability and physiological compatibility compared with isolated supplements, supporting their integration into routine dietary strategies. These considerations highlight that dose, timing, and matrix context are essential determinants of clinical efficacy, bridging mechanistic findings with real-world application. Across muscle, tendon, bone, and neural injury contexts, a consistent translational pattern emerges. For muscle recovery, protein–carbohydrate ingestion (20–40 g protein) within the first 24 h post-exercise enhances mTOR activation and limits proteolysis. In tendon rehabilitation, 15 g gelatin or collagen peptides with vitamin C administered ~60 min before loading optimizes collagen synthesis. For bone health, calcium (1000–2000 mg/day) and vitamin D (800–2000 IU/day) remain essential to reduce stress-fracture risk, particularly in populations with low baseline status. A consolidated synthesis of the evidence discussed across these injury contexts is presented in **Table 1**. Importantly, the table highlights both the reproducible benefits and the variability of response associated with functional-food interventions. While protein-based, collagen-targeted, omega-3, and several polyphenol-rich strategies consistently improve selected biochemical or functional outcomes, other interventions show context-dependent or limited efficacy. This pattern underscores that clinical benefit depends not only on the identity of the nutrient, but also on timing, formulation, baseline nutritional status, injury severity, and the specific physiological demands of the recovery phase.

## 7 Integrative models of nutrition and injury prevention: translational and practical perspectives

Nutrition in the context of sports injury prevention and recovery has evolved from a supportive role to a central biological determinant of resilience and tissue repair. Evidence across muscle, tendon, bone, and neural injury models demonstrates that nutritional factors regulate key molecular processes governing inflammation, oxidative stress, and tissue remodeling. These processes are coordinated through the interaction of mechanical loading, nutrient timing, and metabolic regulation. At the cellular level, bioactive nutrients modulate redox equilibrium via Nrf2 activation, suppress excessive inflammation through inhibition of NF-κB and COX-2, and promote anabolic regeneration by stimulating mTOR and IGF-1 signaling. Translation of these mechanistic insights into clinical practice requires synchronization between nutrient delivery and the biological phases of repair (inflammation, proliferation, and remodeling), ensuring that metabolic resources are available when cellular responsiveness is highest. In practice, nutrient periodization parallels training periodization. During immobilization, maintaining energy intake and protein supply is essential to offset catabolic loss. As inflammation resolves, antioxidant-rich foods and omega-3 fatty acids support the transition toward tissue rebuilding. During rehabilitation, ingestion of collagen or gelatin combined with vitamin C approximately one hour before exercise ensures substrate availability for collagen synthesis during peak fibroblast activity. This phase-specific approach reflects a precision-nutrition framework, in which dietary composition and bioactive components are tailored to injury type, recovery stage, and metabolic demands.

To facilitate clearer interpretation of these multilevel relationships, **Figure 2** summarizes how distinct nutritional inputs converge on a limited set of integrated molecular targets and, in turn, shape physiological and functional recovery outcomes. As illustrated, apparently diverse interventions, including protein-based formulations, collagen-supportive nutrients, polyphenols, omega-3 fatty acids, and neuroprotective compounds, repeatedly influence core signaling pathways related to inflammation, oxidative balance, anabolic regulation, mitochondrial function, and extracellular matrix remodeling. This convergence provides a mechanistic basis for understanding how nutritionally distinct interventions may lead to coordinated improvements in soreness, redox homeostasis, collagen organization, tissue stiffness, neuromuscular recovery, and cognitive function. The integrative perspective of injury recovery can be understood through several overlapping paradigms that converge on shared biological principles. Mechanical strain interacts with metabolic adaptation, forming a mechanobiometabolic feedback system. Nutrients such as omega-3 fatty acids and polyphenols influence membrane properties and focal adhesion signaling, thereby improving mechanotransduction and collagen organization. Muscle, tendon, and bone operate as an integrated continuum, where energy sufficiency and amino-acid availability sustain an anabolic environment across tissues. In parallel, the gut–immune–muscle axis modulates systemic inflammation, nutrient absorption, and immune regulation, shaping recovery capacity. A complementary neuro-energetic perspective highlights that the brain, like muscle, is vulnerable to post-injury energy deficits, with nutrients such as DHA, creatine, and curcumin supporting mitochondrial function and synaptic integrity. Together, these models describe a coordinated biological system in which diet quality, microbiome stability, endocrine balance, and metabolic regulation collectively determine recovery outcomes.

Translating these concepts into practice requires multidisciplinary coordination among physicians, dietitians, physiotherapists, and conditioning specialists. Rehabilitation should begin with assessment of energy availability, micronutrient status, and inflammatory markers to identify barriers to optimal healing. Nutritional strategies should be aligned with the injured tissue and recovery phase, synchronizing nutrient intake with periods of increased perfusion and metabolic activity. Effective implementation depends on athlete adherence and education, with emphasis on whole-food approaches rather than isolated supplementation. Digital monitoring tools may further support this process by linking dietary intake with biochemical and functional outcomes. Preventive nutrition extends beyond injury treatment to reinforce tissue resilience. Regular intake of anti-inflammatory and antioxidant-rich foods, combined with balanced macronutrient distribution, supports nitrogen balance and reduces chronic low-grade inflammation associated with overuse injuries. When integrated with training cycles, these strategies function as preventive interventions, enhancing adaptation while minimizing metabolic stress. Modern high-performance programs increasingly adopt integrated models in which nutrition holds equal importance to load management and psychological conditioning. Advances in wearable technologies and metabolic profiling now enable prediction of injury risk and personalization of nutritional strategies. Future progress depends on refining translational frameworks that bridge mechanistic understanding with real-world application. These models must remain adaptable to diverse injury types, training environments, and individual variability. As evidence continues to evolve, nutrition will increasingly be recognized not only as sustenance but as a targeted therapeutic strategy capable of modulating the molecular architecture of recovery and enhancing long-term resilience.

While preclinical studies provide detailed insights into signaling pathways such as NF-κB, Nrf2, PI3K/Akt, AMPK, and mTOR, translation into clinical practice requires careful consideration of human physiology, bioavailability, and safety. Notably, many animal studies employ doses (e.g., 50–100 mg/kg curcumin) that exceed typical human intake, emphasizing the need for scaled and bioavailable formulations (e.g., nano-curcumin, curcumin–piperine complexes). Current human evidence supports moderate, physiologically achievable doses administered over defined time windows, rather than pharmacological loading. Furthermore, excessive antioxidant supplementation may attenuate exercise-induced adaptations, highlighting the importance of dose optimization rather than maximal intake. Therefore, future research should prioritize well-controlled human trials, standardized dosing protocols, and biomarker-guided interventions to validate the clinical efficacy of polyphenol–exercise strategies. Bridging this gap will enable the transition from mechanistic understanding to evidence-based nutritional prescriptions in oncology and sports rehabilitation. From a pharmacokinetic perspective, the biological efficacy of polyphenols in humans is strongly influenced by their bioavailability and metabolic fate. Most polyphenols, including curcumin, quercetin, and resveratrol, exhibit relatively low oral bioavailability due to limited intestinal absorption, rapid phase II metabolism (glucuronidation, sulfation), and fast systemic elimination (220). As a result, plasma concentrations achieved through conventional dietary intake are often substantially lower than those employed in preclinical models. For instance, curcumin plasma levels typically remain in the nanomolar range after oral ingestion, whereas micromolar concentrations are often used in *in vitro* studies (221). Following ingestion, polyphenols undergo extensive biotransformation in the small intestine and liver, producing conjugated metabolites that may differ in biological activity from their parent compounds. In addition, interactions with the gut microbiota further modify their chemical structure, generating secondary metabolites that can contribute to systemic effects. These processes introduce significant inter-individual variability in response, depending on factors such as microbiome composition, metabolic status, and dietary background (222, 223).

To overcome these limitations, several formulation strategies have been developed to enhance bioavailability. For example, co-administration of curcumin with piperine has been shown to inhibit hepatic glucuronidation and increase systemic exposure (223). Similarly, nano-formulations, liposomal delivery systems, and phospholipid complexes improve solubility and intestinal uptake, allowing lower doses to achieve measurable biological effects (224). The food matrix also plays a critical role, as polyphenols consumed within whole foods may demonstrate improved stability, absorption, and synergistic interactions compared with isolated compounds (225). From a translational perspective, these pharmacokinetic constraints highlight that effective clinical application depends not only on the intrinsic activity of polyphenols but also on their formulation, timing, and delivery context. Moderate, repeated dosing within physiologically relevant ranges is more likely to sustain bioactive metabolite levels than single high-dose interventions. Therefore, future clinical studies should integrate pharmacokinetic profiling with mechanistic and functional outcomes to better define optimal dosing strategies and to ensure that observed biological effects are achievable under real-world conditions. This consideration is particularly relevant in the context of exercise–polyphenol interactions, where circulating concentrations must be sufficient to modulate redox-sensitive signaling pathways activated during physical stress.

## 8 Integrating polyphenols and exercise in cancer prevention, treatment, and rehabilitation linking the injury–recovery–musculoskeletal resilience axis

Polyphenols and physical exercise should not be regarded merely as ancillary interventions; rather, they ought to be conceptualized as an integrated strategy encompassing cancer prevention, therapeutic approaches, and rehabilitative measures. By establishing a connection between these interventions and the injury–recovery–musculoskeletal resilience continuum, this section offers a mechanistic framework elucidating how cancer and its associated therapies provoke tissue damage, inflammation, oxidative stress, and muscle atrophy, as well as how these deleterious effects may be mitigated. It underscores the synergistic roles of polyphenols in modulating the molecular pathways that govern inflammation, mitochondrial functionality, apoptosis, and redox equilibrium, in conjunction with exercise-induced enhancements in anabolic signaling, neuromuscular integrity, and metabolic adaptability. Crucially, this integrated perspective accentuates musculoskeletal resilience as a pivotal determinant of treatment tolerance, functional recuperation, and overall quality of life for individuals afflicted by cancer. By connecting molecular mechanisms to functional outcomes, this section fortifies the translational significance of the review and underpins the formulation of rational, mechanism-based lifestyle interventions aimed at comprehensive cancer management. The integrated effects of polyphenols and exercise across oncological models are summarized in **Table 2**. The available evidence suggests that combined interventions influence cancer-related outcomes through several recurring biological domains, including inflammatory regulation, mitochondrial and redox control, apoptosis/autophagy balance, angiogenesis, neurotrophic signaling, and anabolic–catabolic remodeling. However, these mechanisms do not appear with equal translational strength across studies. Some are repeatedly associated with tissue protection, muscle preservation, cardioprotection, neuroprotection, or quality-of-life improvement, whereas others remain more dependent on tumor model, tissue context, compound formulation, or exercise protocol. **Table 2** therefore provides a structured synthesis of the intervention evidence and helps position the subsequent compound-specific discussion within a more clinically oriented framework. e evidence summarized in **Table 2** indicates that the translational value of combined exercise–polyphenol interventions is strongest when molecular changes are accompanied by functional or tissue-protective outcomes. Inflammatory control, mitochondrial and redox regulation, apoptosis/autophagy balance, and anabolic–catabolic remodeling emerge as the most consistently recurring domains across curcumin, resveratrol, quercetin, catechin, and exercise studies. In contrast, angiogenesis-related markers, miRNA regulation, fibrosis-associated signaling, neurotrophic remodeling, and receptor-mediated pathways are biologically relevant but more model-specific and require further validation in clinically meaningful settings. This distinction is important because cancer rehabilitation requires interventions that preserve function, reduce treatment-related injury, and improve recovery capacity, rather than approaches supported only by isolated molecular-marker changes.

**Table 2:** Mechanistic convergence and translational relevance of combined polyphenol–exercise interventions in cancer-related models.

Population/model	Intervention	Duration	Key findings	Prioritized mechanistic module/biological targets	Translational relevance/limitation	Ref.
Breast cancer mouse model	Curcumin + swimming exercise	Not specified	Stronger tumor inhibition; 445 differentially expressed genes	Calcium signaling, Wnt, PI3K/Akt, and IL-17 pathways; amino sugar, amino acid, and carbohydrate metabolism	Supports broad molecular reprogramming by combined intervention, but clinical relevance is limited by preclinical model and unspecified duration	(226)
40 overweight women after chemotherapy/radiotherapy	Aerobic training ± curcumin 500 mg/day	8 weeks	Reduced hs-CRP, PTX3, BMI, and body-fat percentage; improved quality of life	Inflammatory regulation; PTX3 and hs-CRP modulation; curcumin-mediated control of exercise-associated inflammatory response	One of the most translationally relevant studies because it includes human cancer patients and functional outcomes, although longer follow-up is needed	(227)
Male Wistar rats, muscle tissue	Moderate-intensity aerobic exercise ± nano-curcumin 80 mg/kg/day	4 weeks	RAS and ERK gene expression decreased; combination more effective	MAPK/ERK suppression; RAS/ERK signaling modulation	Suggests anti-proliferative signaling potential, but tissue and cancer-context specificity require further clarification	(228)
Female BALB/c mice with breast cancer treated with doxorubicin	Aerobic exercise ± curcumin supplementation	6 weeks	Caspase-3 decreased and Bcl-2 increased; combined intervention most protective	Apoptosis balance through Caspase-3 inhibition and Bcl-2 upregulation	Relevant to treatment-induced tissue protection, particularly chemotherapy-related injury, but evidence remains preclinical	(229)
Rats with breast cancer treated with doxorubicin	HIIT ± curcumin 100 mg/kg	8 weeks	ERK1/2 and IL-18 decreased; PI3K increased; combination most effective	ERK1/2 suppression, PI3K activation, IL-18 reduction; cardioprotective signaling	Supports potential cardioprotection during chemotherapy, but optimal exercise intensity and curcumin dose require validation	(230)
Female BALB/c mice with breast cancer treated with doxorubicin	Aerobic exercise ± nanomicelle curcumin 100 mg/kg	6 weeks	Aerobic exercise reduced BAX; curcumin decreased BAX and MDA and increased BCL2; combined effect increased BCL2	BAX/BCL2 apoptosis balance; MDA-related oxidative stress modulation	Nano-curcumin may improve tissue-protective effects, but formulation-specific clinical translation remains uncertain	(231)
Female BALB/c mice with 4T1 breast cancer	Aerobic exercise ± nanomicelle curcumin	6 weeks	Exercise decreased CASP3, CASP9, and BAX; curcumin decreased CASP3 and CASP9 and increased BCL2; combined treatment enhanced cardiac protection	Apoptosis regulation through CASP3/9, BAX, and BCL2 pathways	Relevant to cardiotoxicity mitigation, although evidence remains limited to animal models	(232)
Female BALB/c mice with 4T1 breast cancer treated with doxorubicin	Aerobic exercise ± curcumin	6 weeks	No significant changes in GSH, SOD, CAT, or MDA with combination	Limited modulation of hepatic oxidative-stress markers	Important negative finding showing tissue-specific and context-dependent effects; prevents overgeneralization of curcumin–exercise benefits	(233)
Male Wistar rats with glioblastoma	Endurance training ± nano-curcumin 80 mg/kg	4 weeks	miR-21 expression decreased; p53 increased in training + nano-curcumin group	miR-21 downregulation and p53 tumor-suppressor activation	Promising for tumor-suppressive signaling, but clinical translation requires validation in glioblastoma patients	(234)
Male Wistar rats with glioblastoma	Endurance training ± nano-curcumin 80 mg/kg	4 weeks	TGF-β1, TRAF6, and CTGF increased in tumor group and were reduced by AE and AE + nano-curcumin	TGF-β1/TRAF6/CTGF fibrosis and remodeling signaling	Suggests protection against tumor- or treatment-associated cardiac remodeling, but functional cardiac endpoints are needed	(235)
Female BALB/c mice with 4T1 breast cancer	Endurance training ± curcumin	5 weeks	Cancer growth inhibited; miR-126 upregulated; angiopoietin-1 downregulated, strongest in combined group	miR-126 and angiopoietin-1-mediated angiogenesis regulation	Supports anti-angiogenic and tumor-suppressive potential, but remains model-specific	(236)
Female BALB/c mice with 4T1 breast tumors	Endurance training ± curcumin	5 weeks	Tumor growth reduced; Il4 and Stat-6 expression significantly decreased, strongest effect in combined group	IL-4/STAT6 immune-inflammatory signaling	Indicates possible immune-modulatory anti-tumor activity, but clinical immune profiling is required	(237)
Male Wistar rats with brain tumors	Aerobic exercise ± nano-curcumin 80 mg/kg	4 weeks	Behavioral and motor deficits reduced in exercise, nano-curcumin, and combined groups	Neuroprotection through improved cerebral blood flow, neurogenesis, and synaptic plasticity	Relevant to neurorehabilitation and brain-tumor-related functional impairment, but human evidence is lacking	(238)
Male mice with CT26 colon cancer	Resistance training ± resveratrol 50 mg/kg/day	6 weeks	Combined group showed greatest reduction in tumor volume, improved muscle weight, increased mTORC1 phosphorylation, and decreased LC3BII/I ratio	Anabolic–catabolic balance; mTORC1 activation, AMPK modulation, and autophagy inhibition	Highly relevant to cancer cachexia and muscle preservation, but evidence is preclinical and resveratrol bioavailability remains limiting	(239)
BALB/c mice with CT26 colon cancer	Resistance training ± resveratrol 100 mg/kg/day	6 weeks	Combination group showed highest eMHC expression; improved muscle regeneration; no significant change in MyoD or tumor weight	Myofiber regeneration through eMHC expression; partial anabolic remodeling	Suggests regenerative benefit without consistent tumor effect; supports muscle-focused rehabilitation relevance	(240)
Male rats with DMH-induced colorectal cancer	Quercetin 50 mg/kg p.o. ± exercise training	12 weeks	Combination alleviated depressive-like behaviors, reduced neural damage, and restored BDNF, Trkβ, and β-catenin in prefrontal cortex	Neurotrophic signaling through BDNF/Trkβ/β-catenin; inflammatory suppression	Relevant to cancer-related neurobehavioral dysfunction and neurorehabilitation, but human translation remains uncertain	(241)
Male Wistar rats with colon cancer	Intermittent exercise ± quercetin	8 weeks	Exercise improved hippocampal BDNF and CREB; quercetin alone had no significant effect	Exercise-driven BDNF/CREB neuroplastic signaling	Indicates that exercise may be the dominant stimulus for neuroplasticity, with quercetin acting as a context-dependent modulator	(242)
Female BALB/c mice with MC4L2 breast cancer	HIIT ± quercetin 110 mg/kg	6 weeks	Combined treatment significantly reduced tumor expression of TIE-2 and VEGF-A	Anti-angiogenic signaling through TIE-2/VEGF-A suppression	Promising for tumor angiogenesis modulation, but HIIT feasibility and safety require clinical evaluation	(243)
Rats with DMH-induced colon cancer	Quercetin 50 mg/kg + intermittent exercise	8 weeks	Combination significantly increased intestinal Muc5Ac and Muc4 levels compared with other groups	Mucosal-barrier protection through Muc5Ac and Muc4 regulation	Relevant to gastrointestinal protection in colorectal cancer contexts, but human mucosal validation is needed	(244)
Rats with azoxymethane-induced colon cancer	Quercetin ± intermittent or continuous exercise	Not specified	Quercetin alone improved CAT and SOD and reduced MDA; combination with exercise further enhanced effects	Redox balance through CAT, SOD, and MDA modulation	Supports cardioprotective redox modulation, but duration and optimal exercise mode need clarification	(245)
Female BALB/c mice with breast cancer	Aerobic exercise ± quercetin 110 mg/kg	6 weeks	Combination significantly reduced VEGF-A expression; exercise alone had no effect on VEGF-A; TIE-2 unchanged	VEGF-A-mediated angiogenesis suppression	Suggests synergy between quercetin and exercise for angiogenic control, but effects may be marker-specific	(246)
Rats with azoxymethane-induced colon cancer	Quercetin ± interval or continuous exercise	Not specified	Increased cardiac CAT and SOD and decreased MDA; combined therapy more effective than quercetin alone	Cardiac antioxidant defense through CAT/SOD activation and MDA reduction	Supports combined redox-based cardioprotection, but clinical relevance depends on functional cardiac outcomes	(247)
Rats with NMU-induced prostate cancer	Aerobic exercise ± green tea extract	8 weeks	NF-κB reduced with exercise; p53 reduced with exercise, extract, or both; COX-2 unchanged	Inflammatory and tumor-related signaling through NF-κB, p53, and COX-2	Suggests complementary chemopreventive modulation, but pathway responses are not uniformly beneficial or consistent	(248)
Rats with NMU-induced prostate cancer	Aerobic exercise ± hydroalcoholic green tea extract	8 weeks	Green tea extract and combination reduced histological score; exercise partially improved PAB; PSA unchanged	Redox balance and histoprotection through catechin-related antioxidant signaling	Indicates histological improvement despite limited PSA response; supports need for functional and molecular endpoint integration	(249)
Rats with NMU-induced prostate cancer	Aerobic exercise ± hydroalcoholic green tea extract	8 weeks	No significant change in MMP-2/-9 and VEGF; slight decrease in MMP-2 with exercise	Limited modulation of MMP-2/-9 and VEGF-related angiogenesis/metastasis pathways	Important negative or modest finding showing that low exercise or green-tea extract dose may be insufficient for anti-metastatic signaling	(250)

### 8.1 Curcumin

Curcumin and physical exercise independently demonstrate anti-tumor characteristics; however, the interaction of their effects and underlying mechanisms in the context of cancer remains inadequately investigated. Preclinical evidence indicates that their combination exerts stronger anti-tumor effects than either intervention alone. Integrated transcriptomic and metabolomic analyses have revealed extensive molecular reprogramming following combined treatment, including differential expression of hundreds of genes associated with calcium signaling, Wnt, PI3K/Akt, and IL-17 pathways, alongside alterations in amino acid and carbohydrate metabolism (226) (**Table 2**). Clinical evidence further supports this interaction. In breast cancer patients undergoing chemotherapy or radiotherapy, combined aerobic training and curcumin supplementation reduced inflammatory biomarkers (e.g., PTX3) and improved body composition and quality of life, whereas exercise alone increased hs-CRP, an effect mitigated by curcumin (227). The receptor-mediated signaling perspective is also relevant when interpreting combined exercise–curcumin strategies. Exercise can reshape growth factor sensitivity, inflammatory tone, immune-cell activity, and metabolic signaling, while curcumin may influence receptor-proximal and downstream pathways, including EGFR-related signaling, PI3K/Akt, MAPK/ERK, NF-κB, and STAT3. Therefore, reductions in RAS/ERK expression, Akt-related signaling, inflammatory mediators, or angiogenic markers should not be interpreted only as downstream antioxidant effects, but also as evidence of broader modulation of receptor-driven oncogenic and tissue-remodeling networks (157, 159). At the molecular level, both exercise and curcumin modulate key oncogenic signaling pathways. Combined interventions have been shown to downregulate RAS and ERK expression more effectively than either treatment alone, indicating suppression of MAPK/ERK signaling associated with tumor progression (228). In models of chemotherapy-induced toxicity, particularly with doxorubicin, curcumin and exercise demonstrate complementary protective effects. These interventions reduce pro-apoptotic markers such as Caspase-3 and increase anti-apoptotic proteins such as Bcl-2, thereby attenuating tissue damage (229). Similarly, combined protocols restore disrupted signaling pathways, including normalization of PI3K levels and reduction of ERK1/2 and IL-18, suggesting cardioprotective effects against chemotherapy-induced injury (230). Further studies confirm that curcumin, particularly in nano-formulations, enhances antioxidant and anti-apoptotic responses. These effects include reductions in BAX and MDA and increases in BCL2 expression, with the strongest responses observed in combined exercise–curcumin groups (231, 232). However, not all studies demonstrate consistent benefits, as some models report minimal effects on hepatic oxidative stress markers, suggesting tissue-specific responses (233).

Beyond breast cancer models, similar synergistic effects have been observed in other tumor systems. In glioblastoma models, combined interventions downregulate oncogenic miR-21 while upregulating tumor-suppressor p53, indicating modulation of tumor growth and survival pathways (234). In addition, reductions in TGF-β1, TRAF6, and CTGF expression suggest attenuation of tumor-associated fibrosis and cardiac remodeling (235). Curcumin–exercise combinations also influence angiogenesis and tumor progression. These interventions reduce tumor growth, increase miR-126 expression, and suppress angiogenic mediators such as angiopoietin-1, with stronger effects observed in combined treatments (236). Similarly, suppression of Il4/Stat-6 signaling further highlights anti-tumor immune modulation through combined strategies (237). Functional outcomes also improve, particularly in neurological models. Combined aerobic exercise and nano-curcumin significantly reduce behavioral and motor impairments in brain tumor models, indicating neuroprotective synergy (238). Collectively, these findings demonstrate that the combination of physical exercise and curcumin targets multiple interconnected pathways across cancer-related injury and recovery. Exercise primarily enhances anabolic and survival signaling (e.g., PI3K/Akt) while reducing pro-apoptotic mediators (CAS3, CAS9, BAX). Curcumin complements these effects through modulation of oxidative stress, inflammation, and fibrosis, including suppression of NF-κB, IL-17, TGF-β1, TRAF6, CTGF, and ERK1/2 signaling. At the tissue level, these coordinated mechanisms translate into improved mitochondrial function, reduced oxidative damage, and preservation of structural integrity in muscle, cardiac, and neural systems. Importantly, the combination approach mitigates both tumor-induced and therapy-induced damage, supporting functional recovery and enhancing musculoskeletal and systemic resilience.

Despite the breadth of evidence supporting curcumin–exercise synergy, several inconsistencies should be acknowledged. While many studies report reductions in inflammatory and apoptotic markers, others demonstrate limited or tissue-specific effects, particularly in hepatic oxidative-stress outcomes. These discrepancies likely reflect differences in formulation (native vs. nano-curcumin), dosing strategies, and tissue-specific pharmacokinetics. Curcumin’s inherently low bioavailability further complicates interpretation; as systemic exposure may vary substantially across studies even when nominal doses are similar. Moreover, the interaction between exercise intensity and curcumin supplementation appears to be non-linear. Moderate aerobic training generally enhances the anti-inflammatory effects of curcumin, whereas high-intensity or prolonged exercise may induce oxidative stress levels that exceed the buffering capacity of supplementation alone. This suggests that curcumin may be most effective when integrated within controlled training loads rather than extreme physiological stress conditions. Another important limitation is the predominance of preclinical models, in which tumor biology, metabolism, and immune responses differ from human systems. Although transcriptomic and signaling-pathway analyses provide valuable mechanistic insights, their direct translation to clinical oncology remains uncertain. Therefore, while curcumin demonstrates strong potential as a multi-target modulator of inflammation, apoptosis, and metabolic signaling, its clinical effectiveness depends on optimized formulation, dose standardization, and alignment with exercise protocols. Future studies should prioritize human trials that directly compare different curcumin delivery systems and training modalities to clarify its translational applicability.

### 8.2 Resveratrol

Preclinical evidence suggests that the combination of resistance training and resveratrol exerts synergistic effects on cancer-associated muscle wasting. In tumor-bearing models, cachexia is characterized by reduced muscle mass, suppressed mTORC1 signaling, and increased AMPK activation and autophagy (LC3BII/I ratio), reflecting a shift toward catabolic metabolism. Combined intervention restores mTORC1 phosphorylation while attenuating AMPK activation and autophagic signaling, thereby re-establishing anabolic–catabolic balance and limiting muscle degradation. Notably, this approach is also associated with reduced tumor progression compared with sedentary controls. These findings highlight a coordinated mechanism in which exercise-induced anabolic signaling is complemented by resveratrol-mediated metabolic regulation, collectively preserving muscle function and mitigating cachexia (239). Additional studies further support the role of this combination in muscle regeneration. Regenerative markers such as embryonic myosin heavy chain (eMHC) and MyoD have been evaluated to assess myogenic responses. Combined treatment significantly increases eMHC expression compared with exercise alone, indicating enhanced myofiber regeneration and repair. Although effects on tumor mass and MyoD expression are less consistent, improvements in muscle regenerative capacity remain evident. Overall, these results suggest that resistance training combined with resveratrol enhances muscle repair primarily through stimulation of regenerative pathways and partial restoration of anabolic signaling, offering a promising strategy for counteracting cancer-related muscle loss (240).

Although resveratrol demonstrates promising effects on muscle preservation and metabolic regulation in tumor-bearing models, its overall efficacy remains context-dependent. Notably, improvements in muscle mass and anabolic signaling are more consistently observed when resveratrol is combined with resistance training, whereas supplementation alone often produces modest or inconsistent effects. This suggests that resveratrol acts primarily as a potentiator of exercise-induced adaptations rather than an independent therapeutic agent. In addition, conflicting findings regarding tumor progression and muscle regeneration highlight the complexity of its biological actions. While some studies report reduced tumor growth and enhanced mTORC1 signaling, others show no significant change in tumor weight despite improvements in muscle-regeneration markers. These differences may reflect variations in tumor model, resveratrol dosage, and metabolic state of the host organism. Another key limitation is resveratrol’s rapid metabolism and limited systemic bioavailability, which may restrict its physiological impact *in vivo*. As with other polyphenols, the discrepancy between administered dose and active circulating metabolites complicates direct comparison across studies. Collectively, current evidence suggests that resveratrol contributes to muscle preservation and metabolic stability in cancer-associated conditions, particularly when combined with mechanical loading. However, its isolated effects appear insufficient to drive robust anabolic responses, underscoring the importance of integrating nutritional and exercise-based interventions for optimal outcomes.

### 8.3 Quercetin

Preclinical studies indicate that quercetin, particularly when combined with exercise, exerts significant neuroprotective and anti-inflammatory effects in cancer models. Cancer induction is associated with neurobehavioral impairments, including depressive-like symptoms, neuronal degeneration, increased inflammatory cytokines, and reduced expression of neurotrophic factors such as BDNF, Trkβ, and β-catenin. Both interventions, especially in combination, attenuate these alterations by reducing neuroinflammation and restoring neurotrophic signaling. The combined approach enhances the BDNF/Trkβ/β-catenin axis, supporting neuroplasticity and preserving cognitive function without compromising anti-tumor efficacy (241). Complementary evidence highlights the interaction between quercetin and exercise in modulating neuroplasticity. Exercise, either alone or combined with quercetin, significantly increases hippocampal BDNF and CREB levels, whereas quercetin alone shows limited effects. These findings suggest that exercise acts as the primary driver of neuroplastic adaptation, with quercetin potentially enhancing or stabilizing this response (242). Beyond neural effects, quercetin–exercise interactions influence tumor biology. Combined high-intensity interval training (HIIT) and quercetin supplementation significantly reduce the expression of angiogenic markers such as VEGF-A and TIE-2 compared with exercise alone, indicating enhanced anti-angiogenic activity (243). Quercetin also modulates epithelial and barrier-related responses in cancer models. Combined treatment increases intestinal mucosal proteins such as Muc5Ac and Muc4, suggesting improved barrier integrity and mucosal protection under carcinogenic conditions (244). Systemic antioxidant effects are another key component of quercetin activity. Quercetin enhances antioxidant defenses by increasing SOD and CAT while reducing MDA levels, with these effects further amplified when combined with exercise, indicating synergistic cardioprotective activity (245). Similarly, combined aerobic exercise and quercetin reduce VEGF-A expression in tumor tissue more effectively than exercise alone, reinforcing their role in suppressing angiogenesis (246). Consistent findings across models confirm that combined interventions more effectively attenuate oxidative stress compared with either strategy alone (247).

The effects of quercetin supplementation exhibit considerable variability across studies, particularly when evaluated in isolation. While several investigations demonstrate improvements in antioxidant capacity and reductions in inflammatory markers, others report minimal or no significant changes in neuroplasticity-related pathways or functional outcomes. This inconsistency suggests that quercetin’s biological activity may depend strongly on the presence of complementary stimuli, such as exercise-induced metabolic stress. Indeed, exercise appears to play a dominant role in activating pathways associated with neuroplasticity and tissue adaptation, including BDNF and CREB signaling, whereas quercetin alone often fails to induce comparable responses. In this context, quercetin may function as a modulatory agent that enhances or stabilizes exercise-induced adaptations rather than initiating them independently. Additionally, differences in bioavailability, dietary matrix, and co-ingested nutrients may contribute to inconsistent findings. Quercetin absorption is influenced by factors such as glycosylation state and interaction with vitamin C or other flavonoids, which are often not standardized across studies. From a mechanistic perspective, quercetin’s ability to simultaneously modulate oxidative stress, inflammation, and angiogenic signaling provides a plausible basis for its therapeutic effects. However, the heterogeneity of outcomes highlights the need for more controlled comparative studies to define optimal dosing strategies and to determine whether quercetin supplementation offers meaningful benefits beyond those achieved through exercise alone.

### 8.4 Green tea

Preclinical evidence indicates that green tea catechins, particularly when combined with aerobic exercise, modulate key molecular pathways involved in cancer progression and tissue stress. These interventions influence inflammatory and tumor-related signaling pathways, including NF-κB, COX-2, and p53. Cancer conditions are associated with elevated NF-κB activity, reflecting heightened inflammatory signaling, whereas aerobic exercise reduces NF-κB levels, indicating partial attenuation of inflammation. Both exercise and green tea extract modulate tumor suppressor signaling, although responses may vary depending on the pathway assessed. COX-2 levels, for instance, appear less responsive to these interventions (248). Green tea also exerts important effects on oxidative homeostasis in cancer models. Cancer induction disrupts the pro-oxidant–antioxidant balance, increasing oxidative stress and tissue damage. Green tea extract, either alone or combined with exercise, reduces histopathological severity and contributes to restoration of redox equilibrium, although effects on clinical markers such as PSA may be limited (249). In addition to oxidative and inflammatory modulation, green tea influences the tumor microenvironment. Studies examining extracellular matrix remodeling markers report modest or selective effects, with limited changes in MMP-9 and VEGF, and slight modulation of MMP-2 under certain conditions (250). Collectively, these findings suggest that green tea catechins exert multi-target protective effects across the injury–recovery–musculoskeletal resilience continuum in cancer models. Mechanistically, catechins act as potent antioxidants, reducing ROS and restoring redox balance in multiple tissues, including prostate, cardiac, and skeletal systems. In parallel, they modulate inflammatory signaling pathways such as NF-κB and contribute to regulation of tumor-related processes. When combined with exercise, these effects become more pronounced. Although exercise can transiently increase ROS production, its integration with green tea enhances overall antioxidant capacity, limits oxidative damage, and improves tissue recovery. Furthermore, green tea may contribute to extracellular matrix remodeling through partial modulation of MMPs and angiogenic factors such as VEGF, thereby indirectly supporting structural integrity and reducing cancer-associated tissue stress.

Despite the recognized antioxidant and anti-inflammatory properties of green tea catechins, the evidence regarding their efficacy in cancer-related models remains mixed. While some studies demonstrate reductions in oxidative stress and improvements in histopathological outcomes, others report minimal or no significant changes in key tumor-related markers such as VEGF, MMPs, or PSA levels. This variability suggests that the biological effects of green tea may be modest or highly dependent on experimental conditions. One potential explanation lies in the dual role of reactive oxygen species in cancer and exercise physiology. While excessive ROS contributes to tissue damage, moderate ROS generation during exercise is essential for adaptive signaling. Consequently, excessive antioxidant supplementation may attenuate beneficial training adaptations, particularly when administered chronically or at high doses. This highlights the importance of dose optimization and timing when integrating green tea into exercise-based interventions. Furthermore, differences in extract composition, catechin concentration, and administration protocols complicate direct comparison across studies. Green tea preparations vary widely in their content of EGCG and other active compounds, leading to inconsistent pharmacological effects. Therefore, although green tea exhibits potential as a supportive antioxidant strategy, its role within the injury–recovery–musculoskeletal resilience continuum appears to be complementary rather than central. Future studies should focus on defining optimal dosing windows and evaluating the interaction between green tea supplementation and exercise-induced oxidative signaling.

The compound-specific evidence discussed above converges on several clinically relevant mechanistic domains, including inflammatory regulation, mitochondrial and redox control, apoptosis/autophagy balance, anabolic–catabolic remodeling, angiogenesis, neurotrophic signaling, and receptor-mediated tissue remodeling. **Table 3** summarizes these domains across the injury–recovery–resilience continuum and relates them to the major functional outcomes reported in cancer-related models. As indicated in **Table 3**, inflammation control, mitochondrial and redox regulation, apoptosis/autophagy balance, and anabolic–catabolic restoration are the most consistently represented domains across exercise–polyphenol studies. Angiogenesis, miRNA regulation, fibrosis-related signaling, neurotrophic remodeling, and EGFR-related receptor signaling are also represented, but their interpretation remains more dependent on experimental model, cancer type, tissue context, and intervention design. Overall, the evidence supports a network-level interpretation in which combined exercise–nutrition strategies influence cancer-related recovery through coordinated regulation of stress amplification, tissue protection, and functional resilience.

**Table 3:** Translational prioritization of exercise–polyphenol mechanisms across cancer-related injury, recovery, and resilience.

Mechanistic module	Intervention type	Experimental or clinical model	Prioritized molecular pathways	Main biological or functional outcome	Translational relevance
Inflammatory control and redox homeodynamics	Curcumin, nano-curcumin, tart cherry anthocyanins, green tea catechins, quercetin, combined with aerobic or resistance exercise	Exercise-induced muscle damage models; breast cancer patients receiving chemotherapy or radiotherapy; tumor-bearing animal models	NF-κB, COX-2, IL-6, TNF-α, PTX3, Nrf2, SOD, CAT, GPx, MDA	Reduced inflammatory burden, improved recovery kinetics, lower soreness, improved redox balance, and partial preservation of functional performance	Highly relevant to supportive care and rehabilitation, but effects depend on dose, timing, formulation, baseline oxidative stress, and treatment phase
Mitochondrial protection and metabolic remodeling	Exercise combined with curcumin, resveratrol, quercetin, or catechin-rich interventions	Doxorubicin-induced toxicity models; cachexia models; exercise-based rehabilitation models	AMPK, PGC-1α, SIRT1, mitochondrial ROS, ATP production, mitophagy-related quality control	Improved mitochondrial function, reduced oxidative injury, improved metabolic flexibility, and attenuation of treatment-related tissue damage	Clinically relevant for fatigue, cardioprotection, muscle function, and treatment tolerance; human pathway-level validation remains limited
Anabolic–catabolic balance and muscle preservation	Resistance training with resveratrol; protein–carbohydrate nutrition; exercise with polyphenol support	Cancer cachexia and tumor-bearing animal models; muscle injury and rehabilitation contexts	IGF-1/Akt/mTOR, mTORC1, AMPK, autophagy, LC3BII/I, ubiquitin–proteasome activity, MyoD, eMHC	Improved muscle preservation, partial restoration of anabolic signaling, reduced catabolic activation, and enhanced regenerative markers	Directly relevant to sarcopenia, cachexia, and rehabilitation outcomes; translation requires stratification by baseline muscle mass, inflammation, and anabolic resistance
Apoptosis, autophagy, and treatment-induced tissue injury	Curcumin or nano-curcumin combined with aerobic exercise; resveratrol or quercetin with exercise	Chemotherapy-induced cardiac or neural toxicity models; tumor-bearing models	BAX/BCL2 ratio, Caspase-3, Caspase-9, p53, miR-21, autophagy-related markers	Reduced apoptosis, improved tissue preservation, neuroprotective effects, and attenuation of chemotherapy-related injury	Important for cardioprotection and neuroprotection, but evidence remains predominantly preclinical and formulation-dependent
Angiogenesis, fibrosis, and tissue remodeling	Curcumin, quercetin, and exercise-based combinations	Breast cancer, glioblastoma, colorectal cancer, and cardiac remodeling models	VEGF-A, TIE-2, angiopoietin-1, TGF-β1, TRAF6, CTGF, MMP-related remodeling	Reduced angiogenic signaling, lower fibrosis-related marker expression, improved tissue remodeling profile	Promising for tumor microenvironment modulation and treatment-related fibrosis, but clinical relevance requires validation using functional and imaging-based endpoints
Neurotrophic and cognitive resilience	Quercetin or nano-curcumin combined with aerobic exercise	Brain tumor and neurobehavioral cancer models	BDNF, Trkβ, β-catenin, CREB, p53, miR-21	Improved neurobehavioral and motor outcomes, preserved neuroplasticity-related signaling, and reduced neuroinflammation	Relevant to cancer-related cognitive impairment and neurorehabilitation, but evidence is still model-specific
Receptor-mediated signaling and oncogenic remodeling	Curcumin, EGCG, quercetin, luteolin, and other nutraceuticals, with or without exercise	EGFR-related cancer models; NSCLC EGFR-TKI resistance models; breast, prostate, and colorectal cancer models	EGFR phosphorylation, dimerization, internalization, ubiquitination, degradation, PI3K/Akt/mTOR, MAPK/ERK, JAK/STAT, EMT-related pathways	Suppression of receptor-driven proliferation, migration, inflammatory signaling, and treatment-resistance pathways in selected models	Mechanistically important but clinically exploratory; translation depends on achievable tissue concentrations, formulation, pharmacokinetics, and safety validation
Integrated exercise–polyphenol network	Combined exercise and polyphenol-rich nutrition across cancer prevention, treatment support, and rehabilitation contexts	Cross-model synthesis from preclinical and limited clinical studies	NF-κB, Nrf2, AMPK/SIRT1, PI3K/Akt/mTOR, MAPK/ERK, apoptosis/autophagy, mitochondrial homeostasis, EGFR-related remodeling	Potential shift from chronic stress amplification toward coordinated repair, muscle preservation, functional resilience, and treatment tolerance	

## 9 Translational limitations

Although this review emphasizes the potential benefits of exercise and polyphenol-rich functional foods in cancer prevention, treatment support, and rehabilitation, their translation into clinical practice remains constrained by several important limitations. These limitations should be explicitly acknowledged because biological plausibility does not necessarily guarantee clinical efficacy. Exercise and polyphenol-based interventions act within complex physiological contexts shaped by cancer type, treatment phase, inflammatory burden, cachexia or sarcopenia status, baseline nutritional state, gut microbiota composition, fatigue severity, age, sex, endocrine status, and functional capacity. Therefore, the current evidence should be interpreted as promising but not yet sufficient to support uniform clinical recommendations across cancer populations. A central limitation is the imbalance between preclinical and human evidence. A large proportion of mechanistic insights in this field comes from *in vitro* systems and animal models. These studies are valuable for identifying molecular pathways, but they do not fully reproduce the complexity of human oncology settings, where tumor biology, treatment toxicity, metabolic disruption, systemic inflammation, endocrine alterations, and behavioral factors interact dynamically. As a result, the extent to which mechanisms such as NF-κB suppression, Nrf2 activation, AMPK/SIRT1 modulation, EGFR inhibition, or mitochondrial remodeling operate in real-world cancer rehabilitation remains uncertain. A further limitation relates to polyphenol bioavailability and pharmacokinetics. Many polyphenols, including curcumin, resveratrol, quercetin, and catechins, show promising biological activity in experimental models, but their clinical effects may be restricted by poor aqueous solubility, rapid metabolism, short circulating half-life, low tissue availability, and extensive transformation by gut microbiota. Whole foods, extracts, purified compounds, and nanoformulations may therefore produce different biological responses, even when they contain similar parent compounds. Moreover, disease-related anorexia, gastrointestinal toxicity, dysbiosis, chemotherapy, radiotherapy, and altered hepatic or renal metabolism may further modify absorption and tissue exposure in cancer patients.

Exercise-based interventions also show substantial heterogeneity. Existing studies differ widely in exercise modality, intensity, frequency, duration, supervision, progression, and adherence monitoring. In many cases, these parameters are not reported with enough detail to allow replication. Importantly, the biological response to exercise depends strongly on treatment phase, baseline muscle mass, cardiometabolic status, fatigue burden, anemia, neuropathy, pain, cachexia risk, and physical function. Thus, the same exercise program may have different effects in patients undergoing chemotherapy, radiotherapy, hormonal therapy, immunotherapy, or post-treatment rehabilitation. Another major translational limitation is the limited pathway-level validation in humans. Although experimental studies suggest that polyphenols can regulate NF-κB, Nrf2, AMPK/SIRT1, mTOR, MAPK/ERK, EGFR-related cascades, and mitochondrial quality-control pathways, many human studies rely on indirect systemic biomarkers rather than direct assessment of pathway activity in muscle, tumor, immune, or stromal tissues. This makes it difficult to determine whether observed improvements in fatigue, inflammation, muscle function, or recovery are actually mediated by the proposed molecular mechanisms. Safety and context dependency should also be considered. Polyphenols are often perceived as inherently safe because they are food-derived, but high-dose or long-term supplementation may not always be biologically neutral. Excessive antioxidant exposure may interfere with physiological ROS-dependent exercise adaptation, and some bioactive compounds may interact with chemotherapy, radiotherapy, hormonal therapy, anticoagulants, or targeted therapies. Similarly, exercise is generally beneficial, but inappropriate intensity or poor timing during active treatment may worsen fatigue, increase injury risk, or reduce adherence in vulnerable patients. These considerations indicate that exercise–polyphenol strategies should be individualized rather than applied uniformly. Finally, clinical outcome selection remains insufficient. Many studies emphasize biochemical endpoints, oxidative stress markers, or inflammatory mediators, while fewer assess clinically meaningful outcomes such as muscle strength, physical performance, fatigue, treatment tolerance, quality of life, recovery kinetics, recurrence risk, or long-term musculoskeletal resilience. Small sample sizes, short intervention periods, inadequate follow-up, incomplete adherence reporting, and insufficient control of background diet further limit interpretation. These issues are particularly important for combined exercise–polyphenol interventions, where both components must be standardized and monitored to determine whether they act additively, synergistically, or independently.

## 10 Research gaps and future directions

Future research should move beyond proof-of-concept designs toward adequately powered, clinically stratified, and mechanistically informed trials. Such studies should define cancer type, treatment phase, cachexia or sarcopenia status, baseline inflammatory burden, nutritional status, fatigue severity, sex, age, endocrine status, microbiome profile, and functional capacity before intervention. Stratification is particularly important because patients with high inflammatory burden, low muscle mass, poor nutritional status, treatment-induced mitochondrial dysfunction, or altered gut microbiota may respond differently from metabolically stable survivors. Polyphenol interventions should be standardized more rigorously. Future studies should report compound source, formulation, dose, duration, food matrix, co-nutrient composition, timing of intake, circulating metabolites, and, where possible, target-tissue exposure. Trials should distinguish whole-food polyphenol sources from standardized extracts and purified compounds, because these formats differ in absorption, metabolism, and physiological relevance. Pharmacokinetic and pharmacodynamic assessments should be incorporated to clarify whether biologically meaningful concentrations are achieved and whether parent compounds or microbial metabolites are responsible for observed effects. Future studies should explicitly address interindividual variability. Rather than treating variability as statistical noise, trials should evaluate whether gut microbial metabotypes, genetic polymorphisms, sex, age, body composition, baseline diet, and disease status predict response to polyphenol-rich interventions. This approach may help identify responders and non-responders and move the field toward personalized nutrition strategies. In cancer patients, particular attention should be paid to treatment-induced dysbiosis, gastrointestinal toxicity, antibiotic exposure, and altered hepatic metabolism, because these factors may substantially modify polyphenol bioavailability and biological activity.

Sex- and gender-sensitive trial design should also become standard. Future studies should report sex-specific results when sample size permits and should consider hormonal status, menopausal state, androgen deprivation therapy, endocrine therapy, and sex-specific differences in body composition and mitochondrial function. Gender-related factors such as dietary behavior, supplement use, exercise preferences, caregiving burden, and adherence should also be considered where relevant. This would improve the interpretation of heterogeneous responses and help develop more individualized rehabilitation strategies. Exercise protocols should also be described with greater precision. Future studies should specify modality, intensity, frequency, duration, progression, supervision, adherence, safety monitoring, and timing relative to cancer treatment. Exercise prescriptions should be adapted to treatment phase and patient tolerance. Progressive resistance training, aerobic exercise, combined training, mind–body exercise, and low-intensity rehabilitation protocols may have different effects on mitochondrial function, anabolic signaling, fatigue, inflammation, and quality of life. Therefore, future trials should not treat “exercise” as a single intervention, but as a structured and dose-dependent therapeutic stimulus. Future studies should also avoid treating polyphenols as uniformly beneficial antioxidants. Their effects should be evaluated as dose-, timing-, formulation-, and context-dependent modulation of redox-sensitive signaling. In cancer rehabilitation, this distinction is critical because patients may experience chronic oxidative stress from tumor progression, systemic inflammation, chemotherapy, or radiotherapy, while still requiring transient ROS-dependent signals for exercise adaptation. Chronic high-dose antioxidant supplementation may therefore be inappropriate in some settings, particularly if it suppresses adaptive Nrf2, AMPK, PGC-1α, and mitochondrial remodeling responses. Long-term trials should compare whole-food polyphenol sources, standardized extracts, and isolated high-dose supplements while monitoring oxidative damage markers together with Nrf2 activation, NF-κB signaling, mitochondrial biogenesis, muscle function, treatment stage, and exercise responsiveness. Mechanistic validation in human populations should be strengthened. Future studies should integrate molecular endpoints with functional outcomes, including NF-κB and Nrf2 activity, AMPK/SIRT1 signaling, mitochondrial function, IGF-1/Akt/mTOR responsiveness, muscle protein turnover, inflammatory cytokines, oxidative damage markers, and microbial metabolite profiles. These endpoints should be interpreted alongside muscle mass, strength, physical performance, fatigue, treatment tolerance, and quality of life. Importantly, pathway-level measurements should be timed relative to both polyphenol intake and exercise exposure, because transient signaling responses may be missed if sampling is not aligned with intervention kinetics.

Future research should also prioritize receptor-mediated mechanisms, particularly EGFR-related signaling. Studies should determine whether nutraceuticals can influence receptor phosphorylation, dimerization, internalization, ubiquitination, degradation, and downstream PI3K/Akt/mTOR, MAPK/ERK, JAK/STAT, NF-κB, and EMT-related pathways at physiologically achievable concentrations. This is especially important because receptor-mediated signaling links growth factor responsiveness to proliferation, inflammation, angiogenesis, tissue remodeling, and treatment resistance. Therefore, future trials should not rely only on systemic oxidative or inflammatory markers, but should also examine whether bioactive food compounds modulate receptor-proximal and downstream signaling networks in relevant tissues. Future research should evaluate whether exercise and polyphenol-rich nutrition act independently, additively, or synergistically. This requires factorial trial designs comparing exercise alone, polyphenols alone, combined intervention, and usual care. Such designs would help determine whether polyphenols enhance exercise adaptation, blunt physiological redox signaling, or exert benefits only in patients with high baseline inflammatory or oxidative stress. Dose-ranging studies are also needed to distinguish nutritional from pharmacological exposures and to identify thresholds at which benefits plateau or potential interference emerges. Overall, future work should aim to translate mechanistic plausibility into clinically actionable strategies. The evidence reviewed here suggests that cancer progression, anticancer therapy, sports injury, and rehabilitation stressors converge on a limited number of interacting biological modules: inflammation, redox signaling, mitochondrial function, anabolic responsiveness, receptor-mediated remodeling, and regenerative-cell competence. Exercise primarily provides mechanical and metabolic signals that stimulate mitochondrial remodeling, neuromuscular adaptation, and anabolic responsiveness. Polyphenols and other bioactive food components may modulate redox-sensitive, inflammatory, and receptor-mediated signaling, but their effects depend on dose, timing, formulation, bioavailability, microbiome-related metabolism, and disease context. Protein- and collagen-based interventions provide structural substrates for repair, but their benefit depends on whether inflammation, endocrine status, and mitochondrial function permit an anabolic response. Thus, the central translational message is not that each intervention acts on a separate pathway, but that combined exercise–nutrition strategies may be most effective when they shift the entire injury–recovery network from chronic stress amplification toward coordinated repair and musculoskeletal resilience.

## 11 Conclusions

The evidence reviewed here suggests that the benefit of combining exercise with polyphenol-rich nutritional strategies does not arise merely from additive effects, but from their convergence on shared biological processes that govern tissue adaptation and recovery. Across the continuum of cancer prevention, treatment, and rehabilitation, the injury–recovery–musculoskeletal resilience axis provides a useful lens to understand how tumor burden, treatment-related toxicity, inflammation, oxidative stress, and metabolic disruption collectively shape functional outcomes. Within this context, exercise acts as a primary driver of mechanical and metabolic adaptation, whereas polyphenols appear to modulate the biochemical environment in which these adaptive responses unfold. A key implication emerging from this synthesis is that exercise and polyphenols should not be considered as isolated supportive interventions, but rather as context-dependent modulators of tissue resilience whose effectiveness is influenced by timing, dosage, and physiological state. Mechanistically, polyphenols such as curcumin, resveratrol, quercetin, and catechins interact with central signaling pathways, including NF-κB, Nrf2, AMPK, and mTOR, while exercise engages overlapping networks that regulate mitochondrial function, protein turnover, and neuromuscular integrity. When aligned appropriately, these interventions may help attenuate treatment-induced decline in musculoskeletal function and support more efficient recovery. However, it should be emphasized that the current evidence base remains limited in its clinical consistency and translational strength.

In light of these limitations, several research priorities can be identified. Future studies should move toward more standardized intervention designs, clearly defining polyphenol type, formulation, dosage, and timing in relation to exercise and treatment phases. There is also a need for well-powered clinical trials that prioritize functional and patient-centered outcomes, such as physical performance, fatigue, treatment tolerance, and quality of life, rather than relying predominantly on biochemical markers. In addition, the development of biomarker-guided or precision nutrition strategies may help explain inter-individual variability in response, particularly when integrating metabolic, inflammatory, and microbiome-related factors. Addressing these aspects will be essential for translating mechanistic insights into clinically meaningful applications. From a clinical perspective, the current evidence supports a cautious but practical approach. Polyphenol-rich foods or well-characterized supplements may be considered as adjunctive components within individualized rehabilitation and supportive care programs, provided that patient-specific factors, including treatment stage, tolerance, nutritional status, and potential interactions, are carefully evaluated. Importantly, nutritional strategies should be integrated with structured exercise prescription and rehabilitation planning, rather than implemented in isolation. This underscores the need for multidisciplinary coordination among oncologists, dietitians, and rehabilitation specialists to optimize both safety and efficacy. Overall, the integration of exercise and polyphenol-based nutrition represents a promising, biologically grounded approach to preserving functional capacity under oncological stress. Its relevance lies not only in mitigating treatment-related damage, but also in supporting the broader goal of maintaining adaptability and resilience throughout the disease trajectory. Future progress will depend on the ability to refine, standardize, and personalize these interventions so that they can be reliably incorporated into clinical practice.
